# Design, Synthesis, and Characterization of Novel, Subtype-Selective Fluorescent Antagonists Targeting the Nociceptin/Orphanin FQ Opioid Peptide Receptor

**DOI:** 10.1021/acs.jmedchem.5c02707

**Published:** 2025-12-31

**Authors:** George J. Farmer, Julie Sanchez, Annabell Millns, Tamzin Antony, Meritxell Canals, J. Robert Lane, Shailesh N. Mistry

**Affiliations:** † Division of Physiology, Pharmacology and Neuroscience, Medical School, School of Life Sciences, 6123 University of Nottingham, Nottingham NG7 2UH, U.K.; ‡ Division of Biomolecular Science and Medicinal Chemistry, School of Pharmacy, University of Nottingham Biodiscovery Institute, University Park, University of Nottingham, Nottingham NG7 2RD, U.K.; § Centre of Membrane Proteins and Receptors, School of Life Sciences, University of Nottingham, Medical School, Queens Medical Centre, Nottingham NG7 2UH, U.K.

## Abstract

The nociceptin/orphanin FQ opioid peptide receptor (NOPr) is a member of the opioid receptor family under investigation for the treatment of depression, Parkinson’s disease, addiction, and pain. Opioid analgesics such as morphine act through μ-opioid receptor (MOPr) activation but cause MOPr-driven side effects that include respiratory depression, tolerance, addiction, and constipation. Bivalent NOPr/MOPr agonists have been shown to confer effective analgesia with an improved side effect profile. However, the development of new NOPr-targeting drugs is challenged by a paucity of pharmacological tools to characterize NOPr-ligands and visualize receptor expression. We report the design, synthesis, and pharmacological evaluation of the first high affinity small molecule NOPr-targeting fluorescent ligands, based on the antagonist: (2*R*)-1-(phenylmethyl)-*N*-(3-spiro­[1*H*-2-benzofuran-3,4′-piperidine]-1′-ylpropyl)­pyrrolidine-2-carboxamide (**C24**). These ligands display excellent selectivity for the NOPr against MOPr, δ (DOPr), and κ (KOPr) opioid receptors and are effective tracers for competition binding assays to evaluate NOPr-ligand affinity and in live cell imaging to visualize NOPr expression.

## Graphical abstract

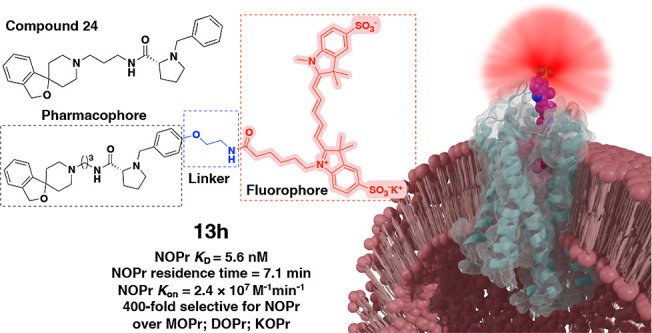

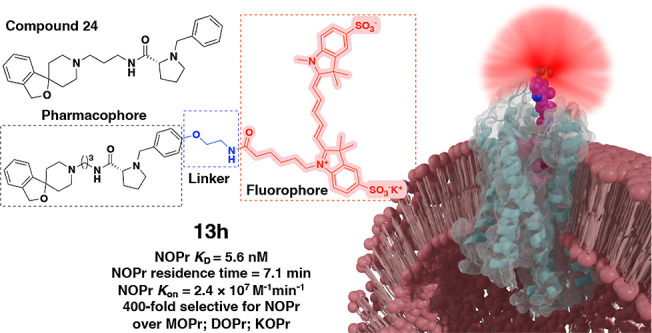

## 1 Introduction

The nociceptin/orphanin FQ opioid peptide receptor (NOPr) is considered a nonclassical member of the opioid receptor family that includes the μ, κ, and δ opioid receptors. While the NOPr shares some structural homology (∼60%) and signaling similarities with the classical opioid receptors, in being a Gi/o coupled class A G protein-coupled receptor (GPCR), it is pharmacologically distinct.^1,2^ The NOPr cannot bind conventional opioid alkaloids like morphine and the promiscuous opioid receptor antagonist naloxone and shows no measurable affinity for other opioid receptor endogenous neuropeptides. Instead, the NOPr displays a highly selective interaction with its endogenous 17-amino acid neuropeptide agonist nociceptin/orphanin FQ (N/OFQ).^3,4^ The NOPr is broadly expressed throughout the body in the brain, spinal cord, and various peripheral tissues,^5^ where it has been shown to regulate physiological processes including emotion, appetite, locomotion, and immune cell response. It also modulates renal, cardiovascular,^6^ and gastrointestinal activity^7^ and has significant effects on nociception, addiction,^8^ and tolerance development.^9^ Given its wide-ranging physiological impact, NOPr is a compelling target for therapeutic intervention. Small-molecule NOPr antagonists are being explored for their ability to enhance motor function in Parkinson’s disease^10^ and as treatments for depression.^11^ ^,^ Conversely, NOPr agonists have shown promise in treating anxiety,^13^ insomnia,^14^ overactive bladder syndrome,^15^ pain and substance use disorders, including blocking the reinforcing effects of cocaine, alcohol, and opioids.^16^ The development of NOPr-targeting analgesics is receiving increasing attention, given their potential to address limitations associated with traditional opioids.^12^

Conventional opioid receptor analgesics, such as morphine, target the MOPr and remain the most effective drugs to treat severe, acute pain. However, adverse effects associated with their MOPr activity, such as tolerance, dependence, and respiratory depression, impact their clinical use. NOPr activation modulates the pharmacodynamic effects of MOPr analgesics, including nociception, addiction, and cardiovascular control, which presents a promising strategy for the development of effective analgesics with improved safety and tolerability profiles.^17,18^ Analgesics with activity at multiple opioid receptors are a promising strategy for opioid painkillers with reduced side effects.^19^ Specifically, activation of MOPr and NOPr pathways simultaneously delivers potent analgesia while alleviating side effects associated with MOPr-selective analgesics across models of acute, inflammatory, neuropathic, and chronic pain.^20,21^ For example, cebranopadol, a MOPr/NOPr agonist that is currently in phase III clinical trials, displays analgesic effects more potent than morphine without traditional opioid-related side effects for the treatment of acute and chronic pain.^22–24^

Despite the therapeutic interest in the NOPr, the highly selective profile and more recent identification of the receptor have contributed to a less comprehensive understanding of NOPr pharmacology compared to the other opioid receptors and a lack of NOPr-selective pharmacological tools. This, in turn, has hindered progress in studying NOPr expression levels, NOPr pharmacology, and, importantly, the discovery of new NOPr-targeting ligands. The NOPr pharmacological toolkit currently available includes radiolabeled N/OFQ,^25–27^ two fluorescently labeled N/OFQ agonists, N/OFQ-ATTO-594 ^28^ and N/OFQ-FITC,^29^ and antibodies that lack adequate receptor selectivity data.

Small molecule fluorescent antagonists offer a promising strategy to expand the NOPr pharmacological tool kit. Unlike agonists, they neither activate the receptor nor cause receptor internalization, enabling the detection of the high affinity (G protein-coupled) and low affinity (G protein-decoupled) states of GPCRs in competition binding experiments. This allows for representative and physiologically relevant affinity measurements to be determined at the NOPr.^30^ Additionally, these properties facilitate the visualization of NOPr expression in whole cells and low-expressing systems and allow microscopy experiments to be conducted at physiological temperatures.^31^

Fluorescently labeled antagonists, when combined with Förster (FRET) or bioluminescent (BRET) resonance energy transfer approaches, can allow binding experiments to be carried out in smaller assay volumes and with greater kinetic resolution, without the safety concerns and costs associated with radiolabeled ligand handling and disposal. Furthermore, fluorescent ligands can facilitate high throughput screening (HTS) with a view to the discovery of novel NOPr-targeting chemotypes. Finally, small molecules can be easily modified, allowing for greater flexibility in experimental design and future fluorescent ligand development.^32–35^

Herein, we report the design, synthesis, and pharmacological evaluation of a library of small molecule NOPr-selective congeners and their fluorescent counterparts, evolved from **C24**, a NOPr antagonist with high affinity (NOPr *K*_i_ = 0.27 nM) and selectivity (*K*_i_ = MOPr, KOPr, DOPr> 2500 nM).^36^ We employed fluorescence confocal microscopy, in vitro binding, and functional assays to pharmacologically characterize these ligands, leading to the discovery of the first, NOPr-selective, small molecule fluorescent antagonists.

## 2 Results and Discussion

### 2.1 Design and Strategy

To underpin our design strategy, we first evaluated the previously published cocrystal structure between **C24** and the NOPr (PDB ID: 4EA3)^37^ (Figure 1a), in order to confirm key ligand–target interactions.

**Figure 1:**
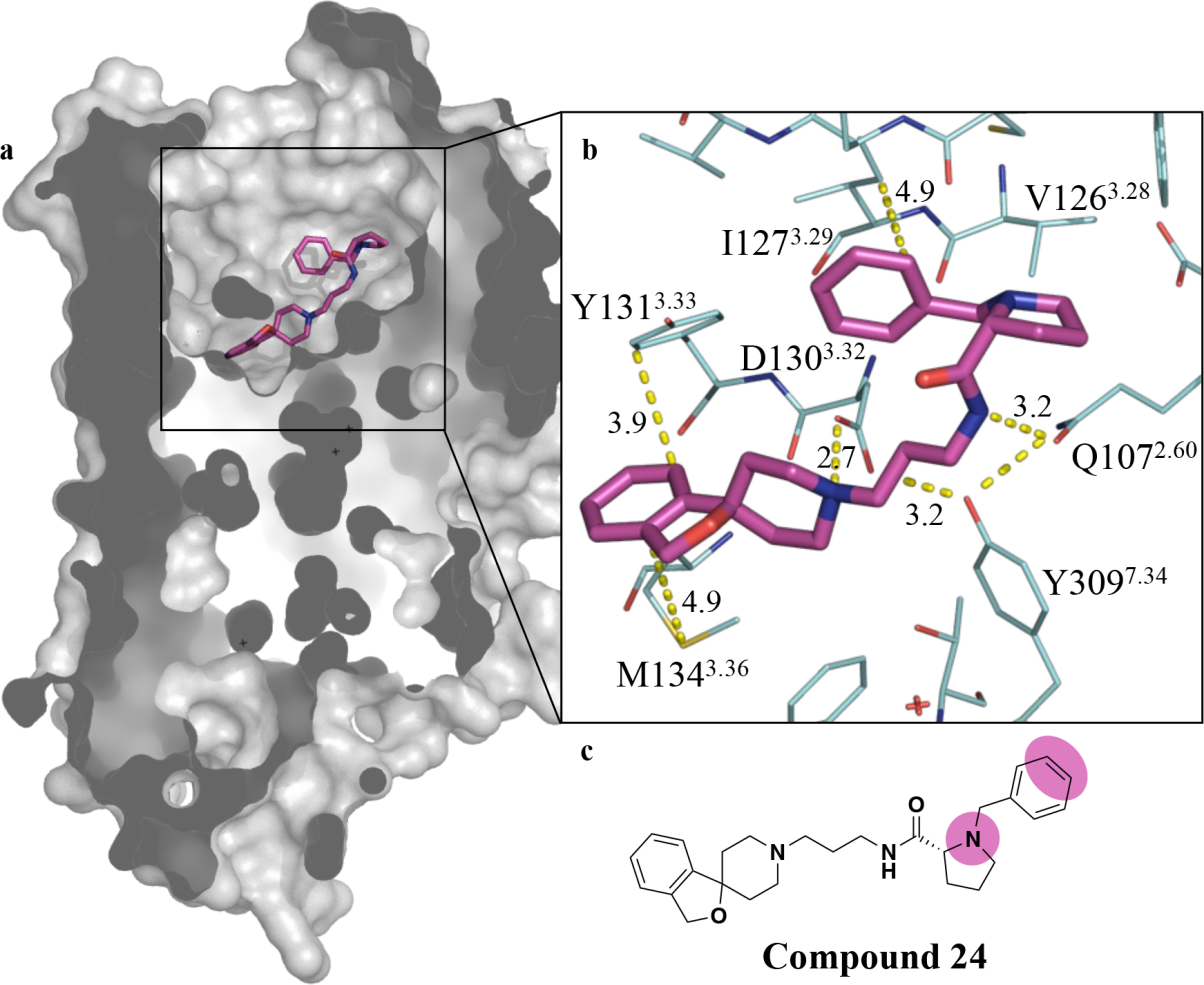
X-ray crystal structure of **C24** bound to the NOPr (PDB ID: 4EA3). (a) Connolly surface representation of the NOPr (gray) with **C24** (magenta stick) in the orthosteric binding site, demonstrating that the bound pose of **C24** projects the *N*-benzyl-d-proline moiety toward the extracellular face. (b) Key receptor–ligand interactions (dashed yellow lines with indicative distances in Å between **C24** (magenta stick) and surrounding amino acid residues (wire representation) annotated using Ballesteros–Weinstein numbering system.^38^ (c) Chemical structure of **C24** highlighting suitable sites for linker attachment (magenta).^37^

The X-ray crystal structure, supported by mutagenesis studies, reveals three key interactions driving the affinity of **C24** for the NOPr. The core spiro­[isobenzofuran-1,4′-piperidine] scaffold binds deep within the binding site (Figure 1a), allowing the isobenzofuran ring system to form a π-stacking interaction with Y131^3.33^ and a hydrophobic interaction with M134^3.36^. The basic piperidino moiety of **C24** forms a salt bridge with D130^3.32^, shown to be crucial for affinity (D130A mutation significantly reduces **C24** binding affinity). Q107^2.60^, stabilized by Y309^7.43^, also forms an important hydrogen bond with the pyrrolidine carboxamide. A Y309A mutation also abolishes the binding of **C24**, highlighting the importance of this stabilizing effect. Finally, the inclusion of the d-prolyl moiety is key for high affinity at the NOPr, as the corresponding *S*-enantiomer displays greatly reduced affinity.^39^ All interactions are summarized in Figure 1b.

Taking this into consideration, we rationalized that an alteration to the d-prolyl amino moiety or the pendant *N*-benzyl group presented suitable sites to attach a linker and ultimately a fluorescent dye (Figure 1c). Linkers are incorporated to navigate a suitable exit vector from the binding site and to incorporate a chemical handle for fluorophore attachment. While ideally, linkers are intended to minimally affect the original pharmacological properties of the parent compound, they can offer opportunities to refine the physicochemical properties of the molecule.^40–42^ The basic amino functionality of the prolyl moiety has been shown to provide superior affinity over other modifications in this region,^36^ as well as offering opportunities to incorporate a linker through either alkylation or acylation. Furthermore, given that **C24** is a peptidomimetic of the NOPr peptide antagonist UFP-101,^37,43^ we sought to explore whether an alanyl linker in place of the benzyl moiety of **C24** would be tolerated or potentially make productive interactions with the receptor. To explore linker length, we also incorporated a β-alanyl linker and generated the corresponding propyl and butyl congeners. Finally, we included linkers retaining the pendant phenyl moiety present on **C24** due to structure–activity relationship (SAR) studies in previous publications, incorporating this region, suggesting it is important for high affinity.^39^ We incorporated an aminoethyl group via an ether linkage to the meta and para positions to facilitate subsequent reaction with an *N*-reactive fluorescent dye. These considerations lead to the synthesis of both *N*-Boc-protected C24 analogues and their corresponding fluorescent ligands described in Section 2.2. We decided to explore the SAR of both *N*-Boc-protected **C24** congeners and their corresponding fluorescent ligands, as previous studies have reported a disconnect between congener and final fluorescent ligand SAR.^44,45^

### 2.2 Synthesis of *N*-BOC-Protected-C24 Congeners **11a-h** and Their Fluorescent Counterparts **13a-h** and **14h**

Of the linkers selected for incorporation into our library synthesis, *N*-Boc-protected amino acids were commercially available for α-alanyl and β-alanyl linkers as well as the corresponding propyl and butyl congeners.^46^ However, linkers bearing benzoyl/benzyl moieties, modified at the meta and para position via an ether linkage to an aminoethyl group, were synthesized as described in Scheme 1.

**Scheme 1:**
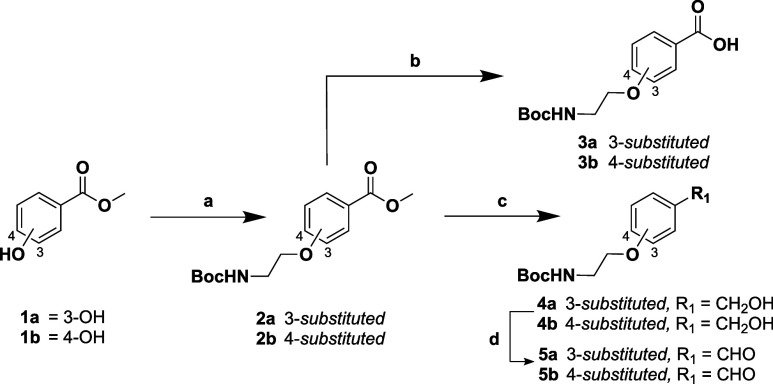
Synthesis of *N*-Boc Protected Linkers (**3a-b** and **5a-b**)­^a^

a Reagent conditions: (a) *N*-Boc-ethanolamine, triphenyl phosphine (TPP), diisopropyl azodicarboxylate (DIAD), tetrahydrofuran (THF), 24 h, 70–81%. (b) LiOH, THF/H_2_O 1:1, 16 h, 89–95%. (c) Diisobutylaluminum hydride (DIBAL-H), 2 h, 84–89%. (d) Dess–Martin periodinane (DMP), dichloromethane (DCM), 16 h, 56–76%.

The Mitsunobu reaction was employed to alkylate methyl-3-hydroxybenzoate (**1a**) and methy-4-hydroxybenzoate (**1b**) with *N*-Boc-ethanolamine, in the presence of Ph_3_P and DIAD in THF. Ph_3_P=O was largely removed from the reaction via precipitation with ZnCl_2_, as reported by Batesky et al., before flash column chromatography on silica gel was performed to give ethers **2a**–**b**. Subsequent hydrolysis with LiOH in THF/H_2_O (1:1), followed by acidification using 2 M HCl, afforded carboxylic acids **3a**–**b**. Compounds **2a**–**b** were also amenable to reduction to the corresponding alcohols **4a**–**b**, which was effected by using DIBAL-H. Subsequent partial oxidation of alcohols **4a**–**b** to the corresponding aldehydes **5a**–**b** was achieved in the presence of DMP.

The general synthesis of **C24** *N*-Boc-protected-congeners (**11a**-**h**) and their corresponding fluorescent derivatives (**13a**-**h** and **14h**) is shown in Scheme 2.

**Scheme 2:**
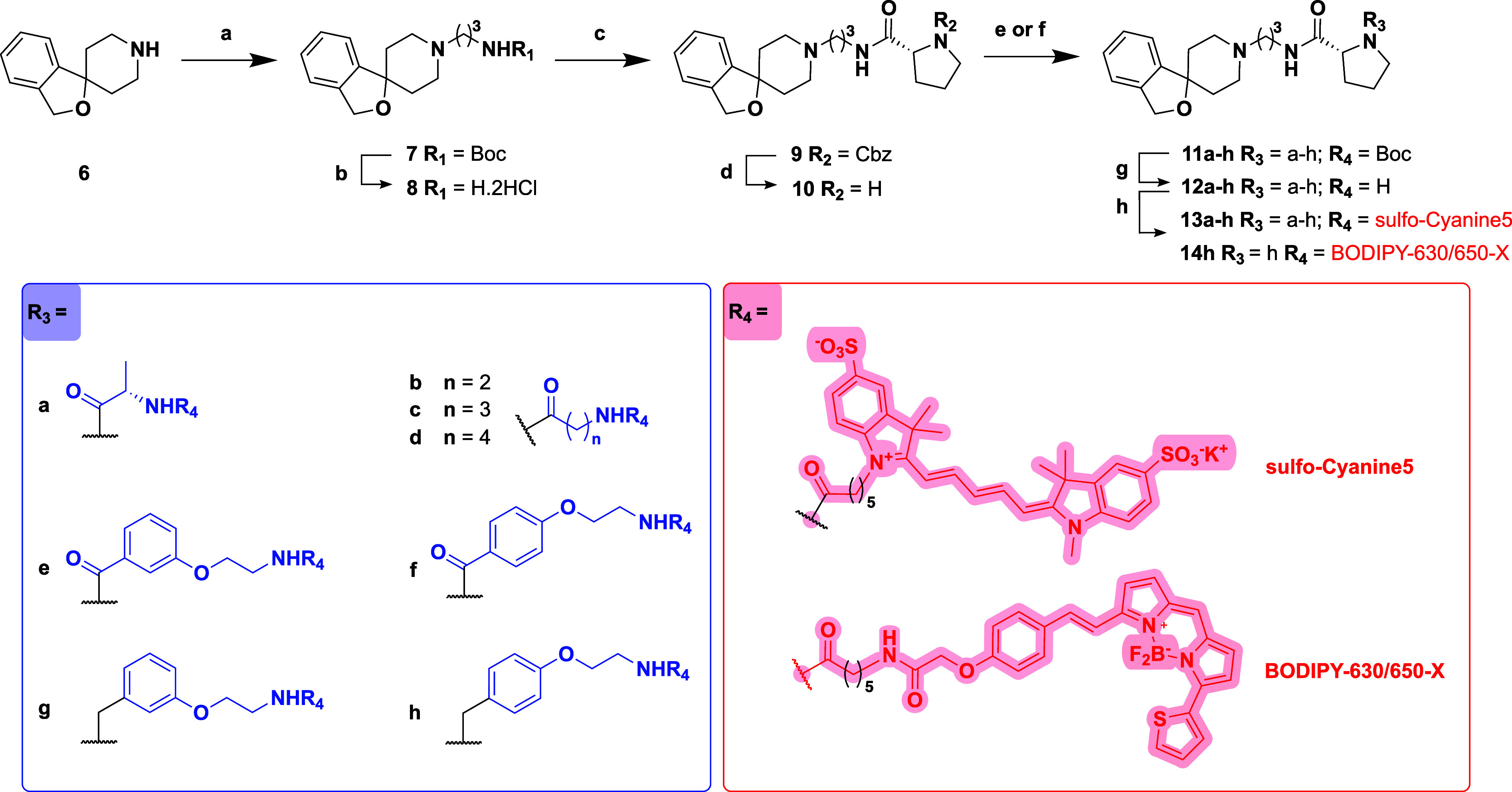
Synthesis of *N*-Boc-Protected-C24 Congeners (**11a-h**) and Their Fluorescent Counterparts (**13a-h** and **14h**)­^a^

a Reagent conditions: (a) *N*-Boc-3-bromopropylamine, *N*,*N*-dimethylformamide (DMF), K_2_CO_3_ 60 °C, 1 h, 84%; (b) 4 N HCl/1,4-dioxane, 3 h, 98%; (c) carboxybenzyl (Cbz)-d-proline, 1-cyano-2-ethoxy-2-oxoethylidenaminooxy)­dimethylamino-morpholino-carbenium hexafluorophosphate (COMU), DMF, diisopropylethylamine (DIPEA), 2 h 95%; (d) 10% Pd­(OH)_2_/C, MeOH, H_2_ atm, 1 h, 100%; (e) Boc-l-Ala-OH, Boc-β-Ala-OH, Boc-GABA–OH, Boc-5-Ava–OH or **3a**–**b**, COMU, DIPEA, DMF, 1 h 50–80%; (f) **5a**–**b**, 2-picoline borane, AcOH, MeOH, 47–60%; (g) 4 N HCl/1,4-dioxane, 100%; (h) sulfonated cyanine5 *N*-hydroxysuccinamide ester (sulfo-Cy5-NHS) or BODIPY-630/650-X-NHS, DIPEA, DMF.

The desbenzyl analogue of **C24** (**10**) was a key intermediate and prepared in 78% overall yield across four steps. This then allowed synthesis of the *N*-Boc-protected-C24 congeners (**11a-h**) and final fluorescent ligands (**13a-h** and **14h**). Following the procedure reported by Trapella et al.,^39^ commercially available 3*H*-spiro (isobenzofuran-1,4′- piperidine) (**6**) was alkylated in DMF with *N*-Boc-3-bromo-propylamine and K_2_CO_3_ to give tertiary amine **7** in 84% yield. Compound **7** then underwent *N*-Boc removal using 4 N HCl/1,4-dioxane to give the corresponding primary amine **8** as a dihydrochloride salt in quantitative yield. After optimizing amide coupling conditions, intermediate **8** was acylated with *N*-Cbz-d-proline using COMU and DIPEA in DMF to afford amide **9**.^48^ Subsequent catalytic hydrogenolysis of the *N*-Cbz group over 10% Pd­(OH)_2_/C afforded the desired intermediate amine **10** in a quantitative yield.

Commercially available *N*-Boc-protected amino acids, Boc-l-Ala-OH, Boc-β-Ala-OH, Boc-GABA–OH, Boc-5-Ava–OH, and recently synthesized linkers **3a**–**b,** were preactivated with COMU in the presence of DIPEA before being coupled with **10**. This afforded the *N*-Boc-protected congeners **11a**–**f** in yields ranging from 20 to 85%. Compounds **5a**–**b** were then used in the reductive amination of **10** in the presence of α-picoline borane, to give **11g**–**h** in moderate yields (61–71%). Protected congeners **11a**-**h** were subsequently *N*-Boc-deprotected with 4 N HCl/1,4-dioxane, affording **12a**–**h** in quantitative yield. Finally, **12a**–**h** were acylated with *N*-reactive far-red emitting fluorophores, 2-[5-[1-[6-(2,5-dioxopyrrolidin-1-yl)­oxy-6-oxohexyl]-3,3-dimethyl-5-sulfoindol-1-ium-2-yl]­penta-2,4-dienylidene]-1-ethyl-3,3-dimethylindole-5-sulfonate (sulfo-Cy5-NHS) (**13a**-**h**) or succinimidyl-6-[2-(p-{(*E*)-2-[4,4-difluoro-5-(2-thienyl)-3a,4a-diaza-4-bora-s-indacen-3-yl]­ethenyl}­phenoxy)­acetylamino]­hexanoate (BODIPY 630/650-X-NHS) (**14h**) in DMF and excess DIPEA to generate a library of nine fluorescent **C24** analogues.

### 2.3 Pharmacological Characterization

#### 2.3.1 Characterization OF *N*-BOC-Protected-C24 Analogues (**11a-h**) and Fluorescent Ligands (**13g-h** and **14h**) in a G-Protein Activation Assay

The functional activity of congeners **11a**-**h** was assessed in a bioluminescence resonance energy transfer (BRET) assay measuring NOPr activation of the Gα_i2_ G protein through release of Gβγ subunits in Flp-In-NOPr-Chinese hamster ovary (CHO) cells, as summarized in Figure 2a.^47^ The purpose of this assay was to determine whether the affinity and antagonist profile were retained after chemical modification of **C24**. To confirm an antagonist profile, we measured the ability of increasing concentrations of **11a**-**h** (1 pM to 10 μM), **13g**-**h,** and **14h** (1 pM to 1 mM) to either stimulate Gai2 activation or to inhibit the action of an EC_80_ concentration of N/OFQ. Inhibition data were used to derive inhibitory potency (pIC_50_) values (Table 1), which are indicative of the relative affinities of the various congeners.

**Figure 2:**
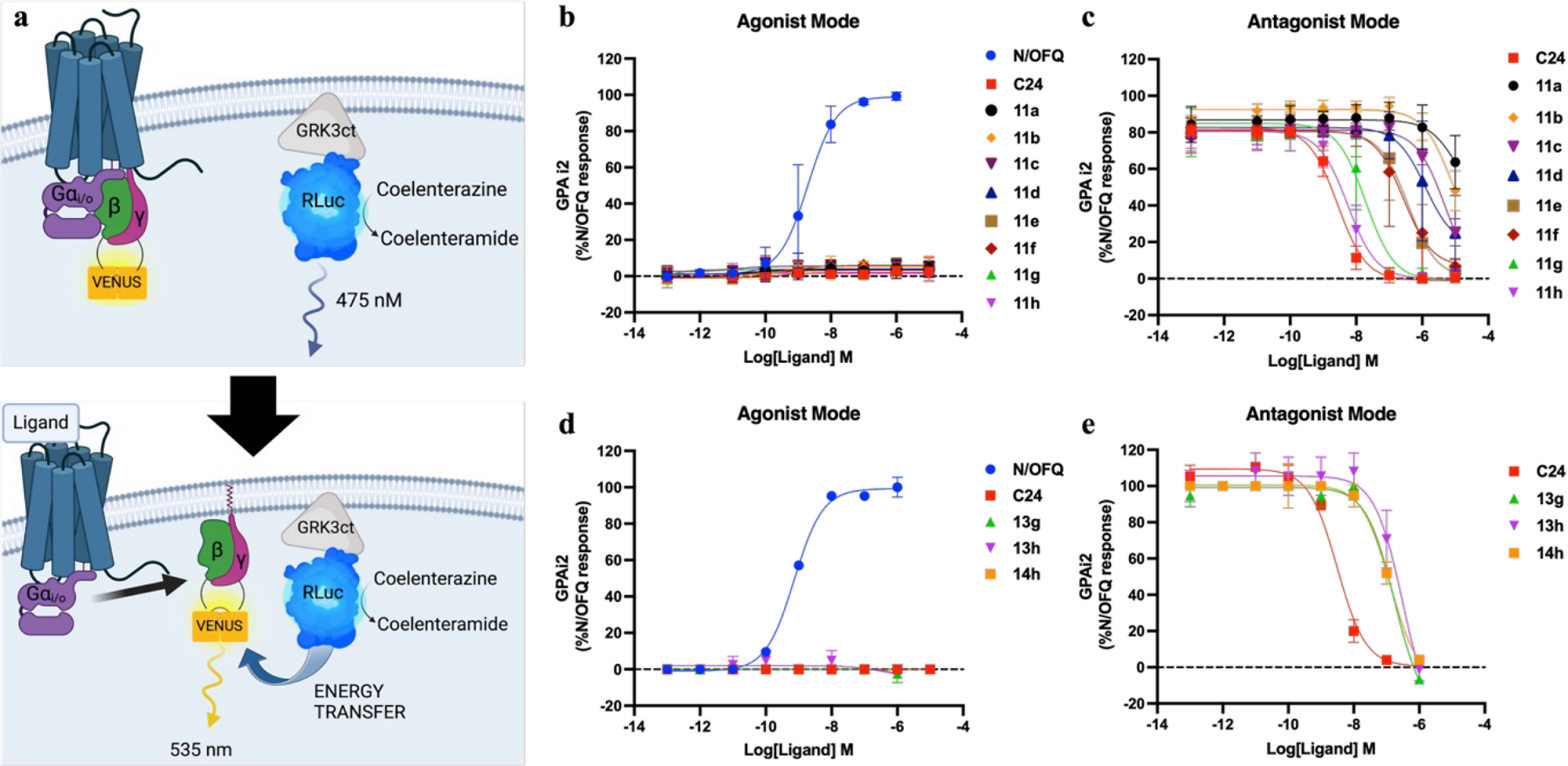
G-protein activation (GPA) assay and corresponding NOPr ligand concentration–response curves for *N*-Boc-C24-congener (**11a**-**h**) and fluorescent-ligands (**13g**-**h** and **14h**). Data represent the mean ± SEM of *n* = 4 experiments performed in duplicate. (a) Illustrative representation of the GPA assay—ligand-induced receptor activation causes G protein dissociation, releasing a Venus-tagged Gβγ (acceptor) to interact with a membrane-localized c-terminus of G protein-coupled receptor kinase 3 (masGRK3ct) fused to a *renilla* luciferase enzyme (RLuc8) (donor). In this assay, **11a**-**h** (10 μM to 1 pM), **13g**-**h,** and **14h** (1 μM to 1 pM) were assessed in agonist mode; NOPr ligands in the absence of the competing agonist N/OFQ and, antagonist mode; NOPr ligands in the presence of a EC_80_ concentration of N/OFQ. After a 10 min incubation with the R-Luc substrate coelenterazine and NOPr ligands, luminescence was measured to quantify GPA as a BRET ratio between acceptor λ_em_ 535 nm/donor λ_em_ 475 nm. Concentration–response curves (b–e) showing GPA by NOPr ligands as a percentage of the maximal response to the endogenous agonist are shown for **11a**-**h** in (b) agonist mode and (c) antagonist mode, and **13g**-**h** and **14h** in (d) agonist mode and (e) antagonist mode.

**Table 1:**
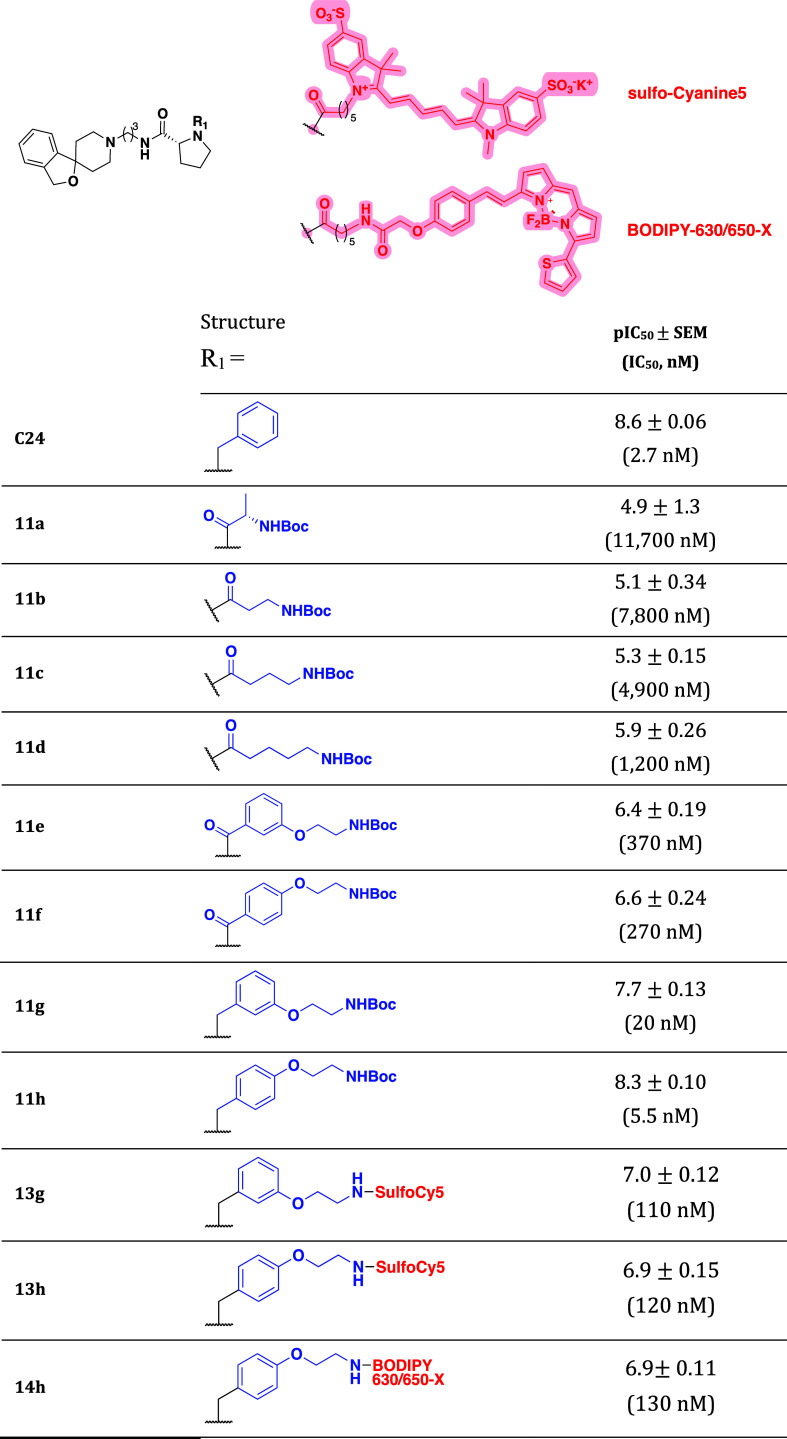
NOPr Inhibitory Potency (pIC_50_) for *N*-Boc-Protected-C24 Congeners (**11a-h**) and Fluorescent Ligands (**13g-h** and **14h**) Compared to C24 in the Functional GPA Assay^a^

a All data represent the mean of *n* = 4 experiments performed in duplicate ±SEM.

Of the ligands tested, regardless of the nature of the linker introduced, no functional agonism at the NOPr was observed for *N*-Boc-C24 congeners **11a**-**h** (Figure 2b) or the corresponding fluorescent ligands **13g**-**h** and **14h** (Figure 2c). The reference compound **C24** exhibited the highest inhibitory potency while congeners **11a**-**h** displayed a clear SAR (Figure 2d). Increasing linker chain length in compounds **11a**–**d** increases inhibitory potency and, by extension, affinity at the NOPr. However, the marked increase in inhibitory potency for compounds **11e**–**h** over **11a-d** reinforces the importance of the phenyl ring of **C24** in driving antagonist affinity.^39^ In comparing **11e**–**f** (benzamide) and **11g**–**h** (*N*-benzyl) analogues, restricting rotation around the phenyl ring via an amide linkage causes an ≥14-fold decrease in inhibitory potency, suggesting free rotation of the benzyl group and basic character of the pyrrolidine ring are important for binding. Additionally, **11e**–**h** show that substitution to the phenyl ring is tolerated, with para-substituted compounds (**11f** and **11h**) exhibiting a higher potency compared to the corresponding meta analogues (**11e** and **11g**) (Table 1). However, this SAR difference is lost after conjugation to far red-emitting fluorophores Sulfo-Cy5 in **13g**-**h** or BODIPY 630/650-X in **14h**. As demonstrated by a ≥5-fold reduction in inhibitory potency for meta-analog when transitioning from **11g** to **13g** and *a* ≥ 20-fold reduction for para-substituted compounds **11h** to **13h** and **14h**, bringing the relative inhibitory potency of all ligands within the comparable range of 109–126 nM.

#### 2.3.2 Whole Cell Imaging Studies to Characterize Binding of Fluorescent Ligands (**13a-h** and **14h**) to the NOPR

To first assess whether the library of fluorescent ligands (**13a-h** and **14h**) can specifically label the NOPr, single time point confocal images of live FlpIn CHO cells stably expressing SNAP-NOPr were captured. Cells were incubated with fluorescent ligands **13a**-**h** and **14h** (1 μM) to assess the fluorescent ligand binding. This was carried out in the presence and absence of the unlabeled NOPr antagonist, SB-612111 (1 μM; NOPr *K*_i_ = 0.33 nM)^49^ (Supporting Table S1), to determine levels of nonspecific labeling.

Previous studies have shown that incorporation of a fluorophore can alter the pharmacology relative to the *N*-Boc-protected precursors, creating a disconnect between congener and fluorescent ligand SAR.^44^ However, in our microscopy experiments, fluorescent ligand binding was consistent with our congener SAR determined in the GPA assay (Section 2.3.1). Binding was observed for fluorescent ligands **13e** and **13f**, with more pronounced binding for compounds **13g** and **13h,** as shown in Figure 3e–h. Minimal binding was observed for **13a**–**d** and representative images are provided in Supplementary Figure S1. For sulfo-Cy5 bearing compounds (**13e**–**h**), where binding was observed, substantial colocalization occurred between the NOPr SNAP-AF488 signal and the far-red-emitting ligands (Figure 3i–l). Additionally, complete displacement of **13e**–**h** by the unlabeled NOPr antagonist SB-612111 (Figure 3m–p) confirmed specific binding to the NOPr orthosteric binding site with minimal nonspecific labeling. In comparison, BODIPY-630/650-X bearing compound **14h** showed significant nonspecific labeling that was unable to be displaced by SB-612111 (Supporting Figure S1). This is likely due to the high lipophilicity of the BODIPY fluorophore, which can lead to nonspecific accumulation in cell membranes, consistent with observations in previous studies.^50–52^ Consequently, only fluorescent ligands demonstrating significant specific binding at 1 μM (**13g**-**h**) were selected for further pharmacological characterization.

**Figure 3:**
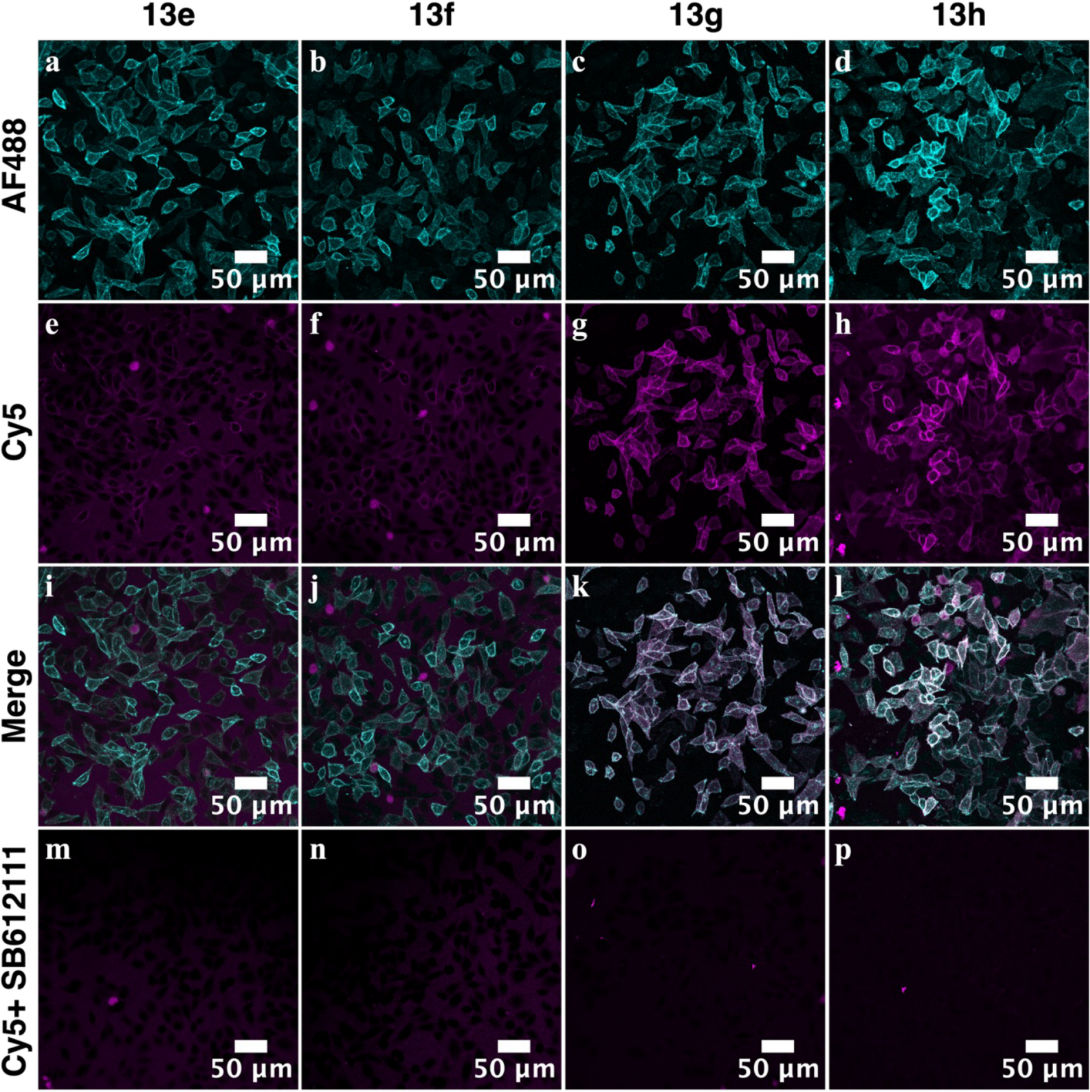
Single time point confocal images of live FlpIn CHO cells stably expressing SNAP-NOPr. Cells were labeled with SNAP-AF488 (cyan) to visualize SNAP-NOPr expression (a–d) and incubated for 30 min at 37 °C with fluorescent ligands **13e**–**h** (1 μM) (magenta) to visualize fluorescent ligand binding (e-h). Colocalization was observed between AF488 and Cy5 channels (i–l), and fluorescent ligand binding was completely displaced by **SB-612111** (1 μM) (m–p), suggesting NOPr-specific labeling.

#### 2.3.3 Affinity and Kinetic Determinations Using Time Resolved-Fluorescence Resonance Energy Transfer

To quantify the affinity and binding kinetics of **13g**-**h** at the NOPr, we used a TR-FRET binding assay to assess the binding of **13g**-**h** in membranes prepared from Lumi4Tb-SNAP-NOPr Flp-In CHO cells. All experiments were carried out in the presence and absence of SB-612111 (10 μM) to determine the nonspecific and total binding, respectively.

##### 2.3.3.1 Saturation Binding Experiments to Determine Fluorescent Ligand Affinity at the NOPR

In order to determine the affinity of the fluorescent ligands at the NOPr, equilibrium saturation binding experiments were carried out using increasing concentrations of fluorescent ligands **13g**-**h** (0.2–400 nM) (Figure 4a,b). The levels of specific binding (total – nonspecific) were calculated and used to determine the dissociation constant (*K*_D_) as a measure of affinity. Specific binding curves are shown in Supporting Figure S2. At the highest concentration tested (400 nM), **13g** and **13h** exhibit saturable binding with minimal nonspecific binding. **13h** demonstrated a 5-fold higher affinity (*K*_D_ = 5.6 nM) than **13g** (*K*_D_ = 19.1 nM), suggesting that modifications at the meta-position are less tolerated than those at the para-position, in line with our congener SAR findings in the GPA assay. Nonetheless, both ligand high affinity and low background signal are promising properties for fluorescence-based studies at the NOPr, enabling low concentrations to be used and improving signal-to-noise ratio. To further investigate the binding characteristics **13g**-**h**, kinetic studies were carried out.

**Figure 4:**
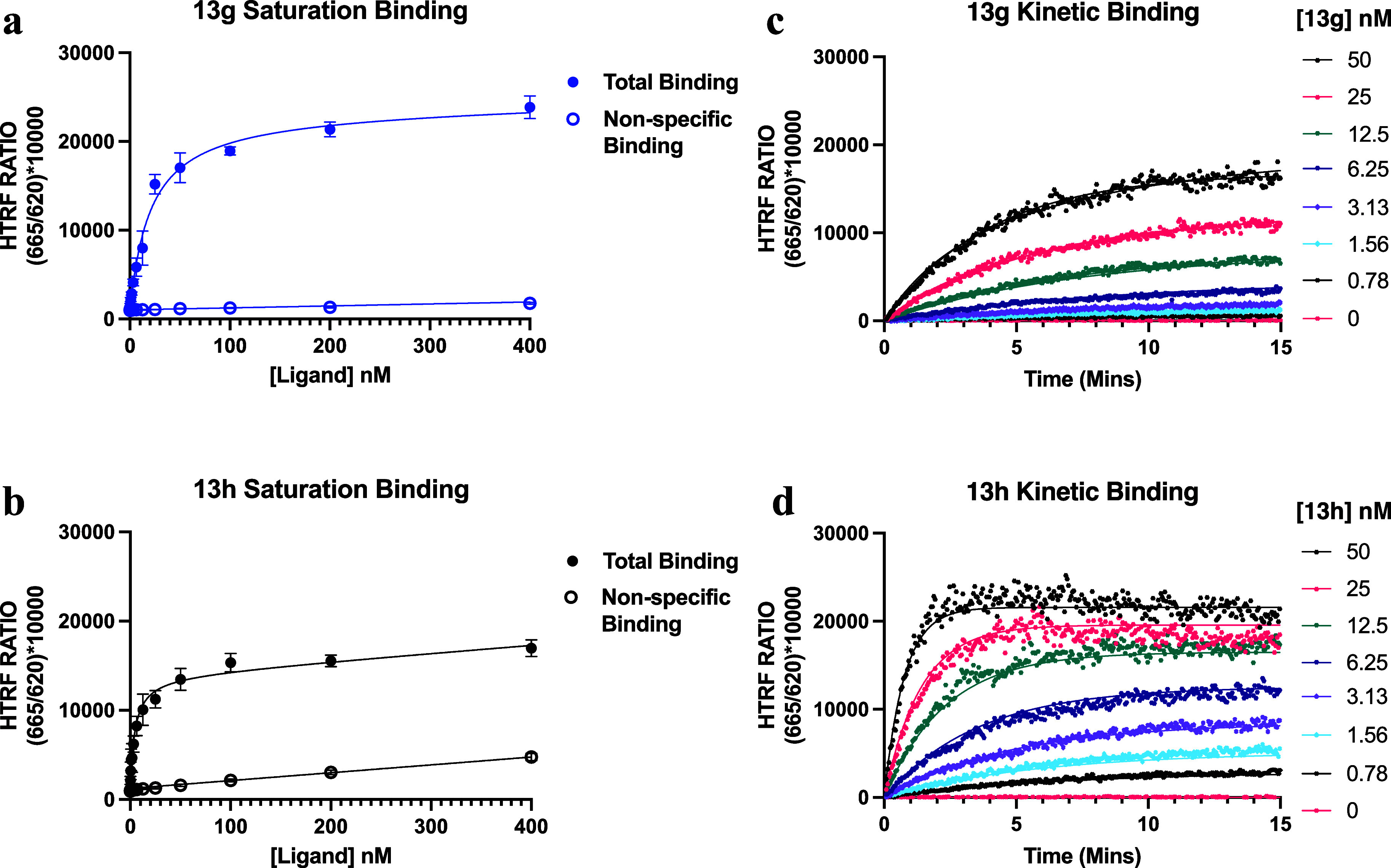
TR-FRET binding experiments to characterize the affinity and kinetic parameters of **13g** and **13h**. End point saturation binding experiments assessing the binding of increasing concentrations (0–400 nM) of (a) **13g** (blue) and (b) **13h** (black) to Lumi4-Tb SNAP-NOPr-CHO membranes at 37 °C to determine fluorescent ligand affinity. For **13g**-**h,** the homogeneous TR-FRET (HTRF) ratio [(acceptor λ_em_ (665 nM)/donor λ_em_ (620 nM) × 10000)] is plotted against ligand concentration (nM) for the total binding (solid circle) and nonspecific binding (NSB) (hollow circle). Kinetic binding of (c) **13g** and (d) **13h** to Lumi4-Tb SNAP-NOPr-CHO membranes was measured over 15 min to determine the NOPr Ligand kinetic parameters in Table 2. For each concentration applied (0–50 nM), the specific labeling (total-NSB) HTRF ratio × 10,000 is plotted against time (minutes). For end point saturation binding assays, data represent the mean ± SEM of *n* = 3 experiments performed in duplicate, and for kinetic binding studies, all data represent the mean of *n* = 3 experiments.

##### 2.3.3.2 Kinetic Binding Experiments

To derive the association rate (*K*_on_) and dissociation rate (*K*_off_) of our ligands, we measured the binding kinetics of increasing concentrations of **13g** and **13h** (0–50 nM) in Lumi4-Tb-SNAP-NOPr-CHO membranes. Measurements were taken over 15 min, until a steady state was achieved, and data were globally fitted to a model to derive parameters provided in Table 2. Kinetic binding over 15 min reveals that differences in ligand affinity are primarily driven by the association rate, with the *K*_on_ of **13h** being >7.7 fold higher than **13g** (Table 2). Compound **13g** (Figure 4c) achieves saturable binding much more slowly than **13h** (Figure 4d) demonstrating that the meta modification reduces the rate of receptor–ligand binding. Despite these differences in *K*_on_, the dissociation rates of both compounds were similar, resulting in comparable receptor–ligand residence times of approximately 10 min. Notably, the kinetically derived *K*_D_ values for both ligands closely align with those determined in saturation binding experiments. Importantly, these interactions are sufficient for high affinity binding, while residence time is not excessively long, making the ligands well-suited as tracers for performing accurate binding experiments and kinetic studies on unlabeled NOPr ligands.^53^

**Table 2:** Affinity and Kinetic Binding Data for Fluorescent Ligands **13g-h** at the NOPr^a^

	**13g**	**13h**
saturation p*K*_D_ (*K*_D_ nM)	7.72 ± 0.12 (19.1 nM)	8.30 ± 0.18 (5.6 nM)
kinetic p*K*_D_ (*K*_D_ nM)	7.45 ± 0.13 (35.7 nM)	8.24 ± 0.14 (6.0 nM)
*K*_on_ (M^–1^ min^–1^)	3.1 × 10^6^ ± 5.8 × 10^5^	2.4 × 10^7^ ± 3.2 × 10^6^
*K*_off_ (min^–1^)	0.10 ± 0.01	0.14 ± 0.02

a Saturation binding data represent the mean ± SEM of *n* = 3 experiments performed in duplicate, and kinetic binding data represent the mean ± SEM of *n* = 3 experiments performed in singlet.

#### 2.3.4 Selectivity Assessment of Fluorescent Ligands **13g-h** Measured in TR-FRET Binding Assays

To understand the selectivity of ligands **13g**-**h** across the opioid receptor family, we performed saturation binding experiments using increasing concentrations of fluorescent ligands **13g**-**h** (0–2 μM) against the NOPr, MOPr, DOPr, and KOPr (Table 3, Figure S3a,b). While some evidence of specific binding to the MOPr and KOPr at higher concentrations was detected, this did not reach saturable levels and thus we were unable to obtain an accurate measure of affinity for these receptors. The ligands in most cases showed similar levels of binding to the nonspecific labeling, demonstrating excellent selectivity for the NOPr.

**Table 3:** Opioid Receptor Affinity of Fluorescent Ligands **13g-h** Naltrexone Based Opioid Red (Cisbio) Determined in TR-FRET-Based Equilibrium Saturation Binding Assays Using Membranes Prepared from Lumi4Tb-SNAP-hNOP, hMOR, hDOR, and hKOR CHO Cells^a^

	*pK*_D_ ± SEM (*K*D nM)			
	NOPr	MOPr	DOPr	KOPr
**13g**	7.72 ± 0.12 (19.1 nM)	n/a	n/a	n/a
**13h**	8.30 ± 0.18 (5.6 nM)	n/a	n/a	n/a
opioid red	n/a	7.60 ± 0.19 (25 nM)	8.05 ± 0.05 (9 nM)	7.59 ± 0.10 (26 nM)

a All data represent the mean ± SEM of *n* = 3 experiments performed in duplicate.

As a positive control to assess binding to Lumi4Tb-SNAP-hMOPr, SNAP-hDOPr, and SNAP-hKOPr membranes, we measured the affinity of a naltrexone-based far-red emitting fluorescent ligand (opioid red (Cisbio), 0–200 nM) (Table 3, Figure S3c). Saturable binding was observed at the MOPr, DOPr, and KOPr, allowing the dissociation constant (*K*_D_) to be determined as a measure of affinity for each of these ORs (Table 3). Notably, there was no measurable affinity of opioid red to the NOPr, in agreement with the published selectivity profile of the parent compound, naltrexone (Table 3, Figure S3c).^54^ The high selectivity for NOPr in membrane preparations highlights the potential of **13g**-**h** as a specific tracer ligand for measurements of affinity at the NOPr and their potential use to selectively label the NOPr and visualize its cellular expression in fluorescence microscopy.

#### 2.3.5 TR-FRET Competition Binding Experiments Using Fluorescent Ligands **13g-h** as Tracers

To assess the application of our fluorescent ligands as tracers in TR-FRET competition binding experiments, **13g**-**h** were used to determine the affinity of unlabeled NOPr ligands (Figure 5). Unlabeled ligands were able to competitively displace **13g**-**h** completely, allowing the determination of inhibitory constant (*K*_i_) values for each ligand after the application of the Cheng–Prusoff equation (Table 4). The ligands tested can be categorized into three groups: antagonists (SB-612111 and C24), full agonists (N/OFQ), and partial agonists (AT-121 and buprenorphine). In our assays, antagonists exhibit similar and, in some cases, higher potencies, while partial agonists demonstrate values consistent with the literature.^21,55–57^ In contrast, the affinity of the full endogenous agonist N/OFQ was approximately 100-fold lower in affinity than previously reported values.^44^ This difference most likely relates to differences in the experimental conditions. In the previous study, the tracer ligand was [^3^H]­N/OFQ, a full NOPr agonist, and binding experiments were conducted in the absence of sodium ions (Na^+^) and guanine nucleotides, conditions which are likely to detect high affinity binding of N/OFQ to the G protein coupled state of the NOPr. In contrast, tracers **13g** and **13h** are antagonists, and our experiments were conducted in the presence of 140 mM Na^+^ ions and 100 μM GppN­(H)­p, conditions that reflect the cellular environment and likely detect N/OFQ binding to the G protein uncoupled, low affinity state.^58,59^

**Figure 5:**
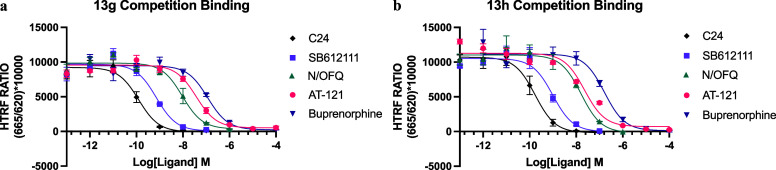
TR-FRET competition binding experiments to determine the affinity of NOPr-targeting ligands. Experiments were carried out in Lumi4-Tb SNAP-NOPr membranes, using fluorescent ligands (a) **13g** (5 nM) and (b) **13h** (25 nM) as tracers to study the NOPr ligands: C24 (0.1 μM to 0.1 pM), SB-612111 (0.1 μM to 0.1 pM), N/OFQ (1 μM to 0.1 pM), AT-121 (100 μM to 0.1 pM), and buprenorphine (100 μM to 0.1 pM). All ligands were able to displace **13g**-**h** completely, allowing the determination of inhibitory constant (*K*_i_) values for each ligand after the application of the Cheng–Prusoff equation, as displayed in Table 4. The data shown represent the combined mean ± SEM of *n* = 3 experiments performed in duplicate.

**Table 4:** Affinity Data for NOPr-Targeting Ligands from Literature Values and TR-FRET Competition Binding Experiments Using Tracers **13g** and **13h** ^a^

	literature *K*_i_ (nM)	**13g**—*K*_i_ ± SEM (nM)	**13h**—*K*_i_ ± SEM (nM)
C24	0.24 ^55^	0.06 ± 0.02	0.09 ± 0.0008
SB-612111	1.3 ± 0.23 ^56^	0.33 ± 0.04	0.43 ± 0.008
N/OFQ	0.08 ± 0.03 ^57^	4 ± 6	8 ± 8
AT-121	3.67 ± 1 × 10 ^21^	17 ± 3	11 ± 10
buprenorphine	77.4 ± 16.1 ^57^	53 ± 7	82 ± 28

a All Data Represent the Mean ± SEM of *n* = 3 Experiments Performed in Duplicate.

#### 2.3.6 TR-FRET Saturation and Competition Binding Experiments Using Fluorescent Ligands 13H in the Presence and Absence of Sodium IONS and Guanine Nucleotides

To investigate the effect of Na^+^ and guanine nucleotides on competition binding experiments, as discussed in Section 2.3.5, we decided to perform competition binding experiments in the presence (Figure 6a) and absence (Figure 6b) of 100 mM Na^+^ ions, which are known to be a negative allosteric modulator of many GPCRs^60,61^ and a nonhydrolyzable form of guanine triphosphate, Gpp­(NH)p (100 mM).^62^ To maintain an equivalent ionic strength in the Na^+^ free condition, 100 mM of the organic cation *N*-methyl-d-glucamine (NMDG) was included in the buffer. Saturation binding experiments were carried out to determine the dissociation constant (*K*_D_) of **13h** in each buffer (Supporting Figure S4, Supporting Table S2). We assessed the ability of the NOPr antagonist SB-612111 and the full NOPr-agonists, endogenous peptide N/OFQ and small molecule MCOPPB, to competitively displace **13h**. All ligands completely displaced **13h**, allowing the determination of their affinity (*K*_i_ values) using the Cheng–Prusoff equation (summarized in Table 5).

**Figure 6:**
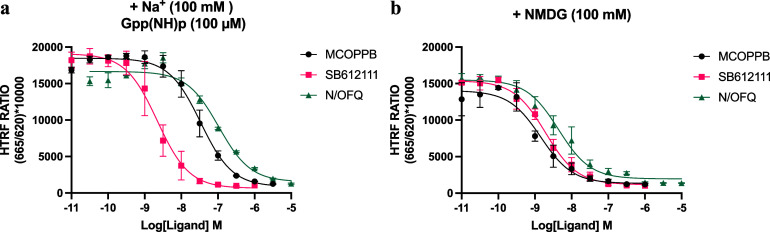
TR-FRET competition binding experiments to determine the affinity of NOPr-targeting ligands in the presence and absence of Na^+^ and Gpp­(NH)­p. Experiments were carried out in Lumi4-Tb-SNAP-NOPr-CHO membranes, and measurement was taken after 2 h incubation at 37 °C. **13h** (10 nM) was used as a fluorescent tracer to assess the binding of increasing concentrations of NOPr ligands: MCOPPB (3 μM to 10 pM), SB-612111 (10 μM to 10 pM), and N/OFQ (10 μM to 10 pM) in buffers containing (a) sodium and Gpp­(NH)p and (b) **NMDG**. All ligands were able to competitively displace **13h** completely, allowing the determination of inhibitory constant (*K*_i_) values (Table 5) for each ligand after the application of the Cheng–Prusoff equation using the representative *K*_D_ of **13h** in each buffer (Supporting Figure S4). The data represent the combined mean ± SEM of *n* = 3 experiments performed in duplicate.

**Table 5:** Affinity (*K*_i_ Values) for NOPr-Targeting Ligands Determined in the Literature and TR-FRET Competition Binding Experiments Using Tracers **13g** and **13h** in the Presence and Absence of Sodium and Gpp­(NH)­p^a^

	Literature *K*_i_ (nM) ± SEM	Na^+^ + Gpp(NH)p *K*_**i**_ (nM) ± SEM	+NMDG *K*_**i**_ (nM) ± SEM
MCOPPB	0.09 ± 0.002 ^63^	15 ± 3	0.9 ± 1
**SB-612111**	1.3 ± 0.23 ^56^	0.9 ± 0.2	1.2 ± 2
N/OFQ	0.08 ± 0.03 ^57^	45 ± 6	2.8 ± 8

a All data represent the mean of *n* = 3 experiments performed in duplicate ±SEM.

The *K*_D_ of fluorescent ligand **13h** in the Na^+^ and Gpp­(NH)p condition (*K*_D_ = 8 nM) was approximately 2-fold higher than in the NMDG condition (*K*_D_ = 17 nM). When assessing agonist binding (N/OFQ and MCOPPB) at the NOPr using **13h** as a tracer, the presence of Na^+^ and Gpp­(NH)­p produced a ∼16-fold decrease in apparent affinity (*K*_i_) when compared to the NMDG condition. Additionally, the determined affinity for the NOPr antagonist (**SB-612111**) showed little change in either condition (∼1.3-fold).

This illustrates that the choice of tracer ligand and assay conditions can be very important for the determination of affinity. For example, when assessing selectivity across receptor subtypes, it is logical to use an antagonist tracer, as they are less sensitive to the receptor conformational state, allowing for more consistent comparisons of ligand affinity across receptor family members.

#### 2.3.7 Fluorescence Microscopy to Determine **13g-h** Opioid Receptor Selectivity

To further assess the ability of **13g**-**h** to selectively label the NOPr in microscopy applications, human embryonic kidney (HEK) 293T cells were transfected with SNAP-hNOPr, SNAP-hMOPr, SNAP-hDOPr, or SNAP-hKOPr and a nuclear localization sequence, cyan fluorescent protein (NLS-CFP). Receptors were labeled with SNAP-AF488 to visualize receptor expression and treated with fluorescent ligands **13g**-**h** at a concentration of 20× their NOPr *K*_D_, as determined in saturation binding experiments. To determine levels of nonspecific labeling, experiments were carried out in the presence and absence of a competing unlabeled antagonist (SB-612111 for NOPr and naloxone for MOPr, DOPr, and KOPr). All receptors showed cell surface expression, as seen by SNAP-AF488 labeling (Figure 7a–d,i–l), and no labeling of MOPr, DOPr, and KOPr was observed for either ligand (Figure 7f–h,n–p). Selectivity was quantified for each ligand by normalizing the fluorescence intensity to the number of cells per image as determined by NLS-CFP expression (Figure 7q–r). The normalized result was adjusted by deducting the intensity measured in the presence of antagonist (SB-612111 for NOPr and naloxone for MOPr, DOPr, and KOPr), effectively removing the contribution of unbound ligand in solution. No significant differences were observed between **13g**-**h,** and results are shown graphically in a bar chart for ligands **13g** (Figure 7s) and **13h** (Figure 7v).

**Figure 7:**
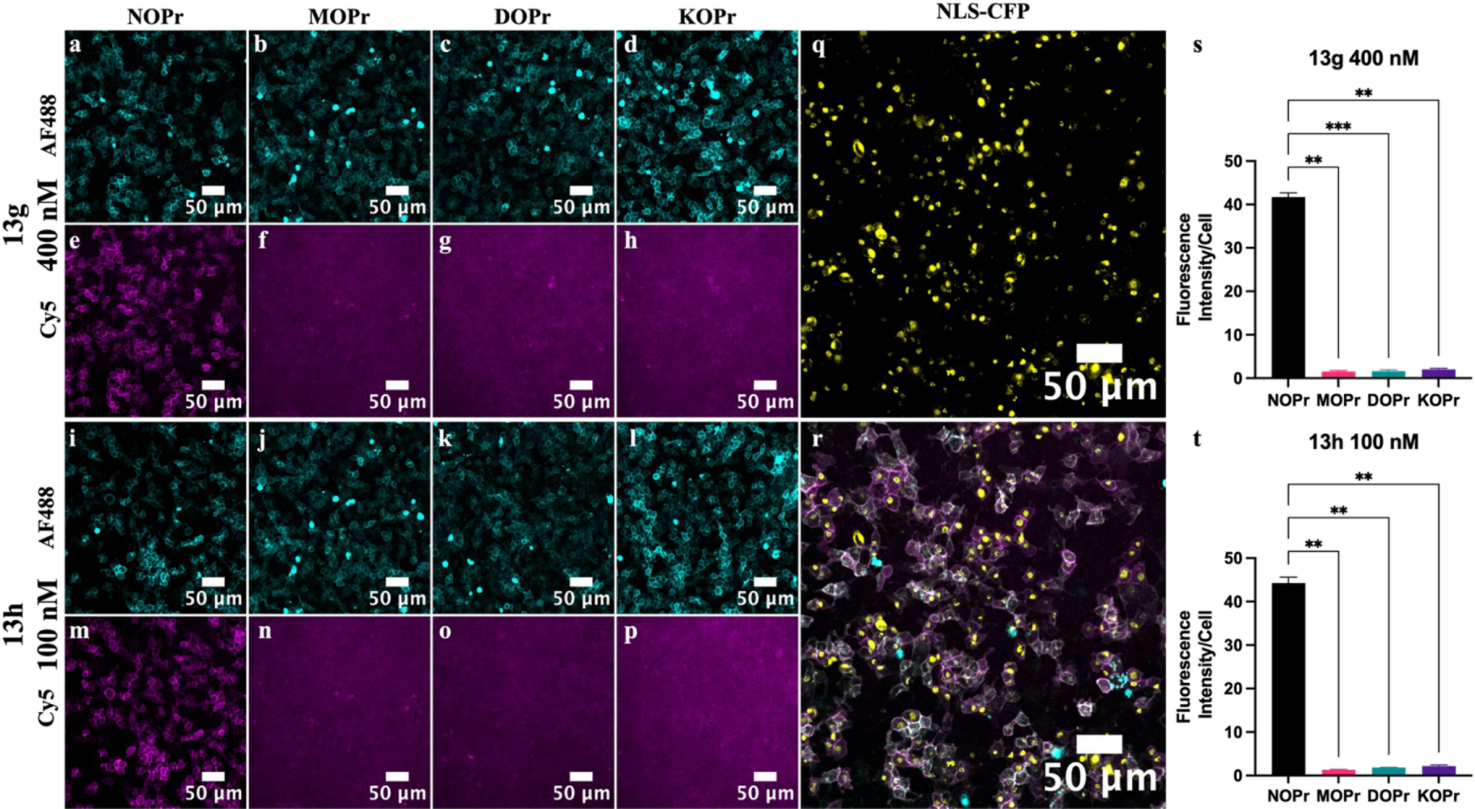
Single time point confocal images of live HEK 293T cells, transiently transfected with SNAP NOPr, MOPr, DOPr, or KOPr and a nuclear marker NLS-CFP. Receptors have been labeled with SNAP-AF488 (cyan) to visualize receptor expression (a–d) and (i–l) and preincubated with fluorescent ligands (magenta) **13g** (400 nM) (e–h) and **13h** (100 nM) (m–p) to observe receptor–ligand binding. To quantify fluorescent ligand selectivity, cells were counted using the nuclear marker NLS-CFP (yellow) (q) and (r), and the fluorescence intensity/cell is represented in bar charts for **13g** (s) and **13h** (t) to demonstrate opioid receptor selectivity. All data represent the mean of *n* = 3 experiments performed in duplicate ±SEM. Statistical analysis was done using repeated measures one-way ANOVA with Dunnett’s multiple comparisons. Asterisks indicate significant selectivity of **13g**-**h** for the NOPr (*p* < 0.05).

## 3 Conclusion

In this study, we describe the design, synthesis, and pharmacological evaluation of a small library of fluorescent ligands based on the high affinity NOPr antagonist, **C24**. Our pharmacological characterization of eight congeners (**11a**-**h**) bearing *N*-Boc-protected linker moieties in place of the pendant *N*-benzyl group of **C24** revealed that retention of the *N*-benzyl pendant is crucial for potent antagonism. Modification of this benzyl moiety is tolerated, with para-substitution being preferred. Conjugation of the *N*-Boc congeners to far-red-emitting fluorophores (sulfo-Cy5 or BODIPY 630/650-X) afforded fluorescent ligands **13a**-**h** and **14h**, whose relative pharmacological profiles were consistent with their corresponding *N*-Boc precursors. Among these compounds, **13g**-**h** emerged as promising fluorescent ligands, demonstrating low nanomolar affinity (19.1 and 5.6 nM, respectively), excellent selectivity over the other opioid receptors (>100-fold for **13g** and >490-fold for **13h**), and favorable kinetic profiles for ligand binding studies. TR-FRET binding experiments demonstrated their utility as tracers for competition binding studies at the NOPr. As such, **13g**-**h** can be useful tools with which to measure affinity and ligand binding kinetics as part of future efforts to develop novel NOPr ligands. Imaging studies confirmed the utility of **13g**-**h** in specifically and selectively labeling NOPr expression in living HEK 293T and CHO cells. As such, these probes may be used to identify and quantify native NOPr expression and distribution in cells and tissues.^64,65^

## 4 Experimental Section

### 4.1 Synthesis

#### 4.1.1 Materials and Equipment

Chemicals and solvents were obtained from Fisher Scientific UK, Sigma-Aldrich, Merck Millipore, Lumiprobe, and Fluorochem and used without purification. All reactions were monitored by thin-layer chromatography (TLC) or liquid chromatography mass spectrometry (LCMS). TLC was carried out using Merck Silica Gel 60 Å F254 plates and examined under UV light (254 and 366 nm), followed by staining with ninhydrin or iodine. LCMS was carried out using a Shimadzu UFLCXR HPLC system coupled to an Applied Biosystems API 2000 LC/MS/MS instrument (ESI). UV detection was at 220 and 254 nm, and the system was fitted with a Phenomenex Gemini-NX 3 μm-110 Å C18, 50 × 2 mm column operating at 40 °C with a flow rate of 0.5 mL/min. Solvent A contained water with 0.1% formic acid, and solvent B was acetonitrile with 0.1% formic acid. The program consisted of 1 min at 5% B; 5–98% B over 2 min, 98% B for 2 min, 98–5% B over 0.5 min, and then 5% for 1 min (Method A). For all compounds, the calculated (calcd) mass and found mass are shown. Purification was carried out using column chromatography using technical grade silica gel from Aldrich, pore size 60 Å, mesh particle size 230–400, and particle size 40–63 μm or Advion Interchim puriFlash SI-HP columns on a Biotage SP4 flash chromatography system. NMR spectroscopy was performed using a Bruker AV­(III) HD 400 NMR spectrometer equipped with a 5 mm BBFO + probe, recording ^1^H and ^13^C NMR at 400.25 and 100.66 MHz, respectively. NMR data were processed using MestReNova (version 14.2.2). Solvents used for NMR analysis were CDCl_3_, (δ_H_ = 7.26 ppm, δ_C_ = 77.16 ppm). DMSO-*d*_6_ was supplied by Sigma-Aldrich (δ_H_ = 2.50 ppm, δ_C_ = 39.52 ppm) and MeOD (δ_H_ = 2.50 ppm, δ_C_ = 39.52 ppm). Chemical shifts (δ) are reported as values in parts per million, and coupling constants (*J*) are given in hertz (Hz). Multiplicities are abbreviated: s, singlet; d, doublet; t, triplet; q, quartet; m, multiplet; and br, broad. Phenomenex C18 onyx monolithic columns were used for small scale RP-HPLC purifications (semipreparative 100 × 10 mm) and analytical HPLC analysis (100 × 4.6 mm). A Shimadzu systems controller SCL-40, degassing unit DGU-405, solvent delivery module LC-40D XR, auto sampler SIL-40C XR, column oven CTO-40C, and photo diode array detector SPD-M40 were used. Solvent A contained water with 0.1% formic acid, and solvent B was acetonitrile with 0.1% formic acid. All pharmacologically tested compounds are >96% pure by HPLC analysis. The analytical program at a flow rate of 1.0 mL min^–1^ consisted of 1 min at 10% B 10–95% B over 11 min, 95% B for 2 min, 95–10% B over 1 min, and then 10% B for 1 min (Method B). UV detection was at 254 nm, max plot 200–800 nm, and additionally 320 nm for red-shifted fluorescent ligands. HRMS was carried out with a Bruker microTOF mass spectrometer using ESI-TOF operating in positive or negative ion mode.

#### 4.1.2 General Method **1**

##### 4.1.2.1 Hydroxybenzoate Alkylation with *N*-Boc-Ethanolamine via a Mitsunobu Reaction

To a solution of the corresponding hydroxybenzoate **1a** or **1b** (1.0 equiv) and Ph_3_P (1.5 equiv) in THF (2 mL), *N*-Boc-ethanolamine (1.5 equiv) in THF (8 mL) was added. The mixture was stirred at 0 °C under a nitrogen atmosphere before DIAD (1.5 equiv) was added dropwise. The reaction was allowed to warm to RT and stirred for 24 h until completion, confirmed by TLC and LCMS. The solvent was then removed in vacuo, and the TPPO side product was removed via filtration after precipitation with ZnCl_2_ in Et_2_O. The filtrate was dried in vacuo, and the resulting residue was purified using column chromatography as described.

#### 4.1.3 General Method **2**

##### 4.1.3.1 Basic Ester Hydrolysis of Benzoates **2a** and **2b** to Corresponding Benzoic Acids **3a** and **3b**

To a solution of the corresponding methyl ester **2a** or **2b** (1.0 equiv) and LiOH (5.0 equiv) in THF (2 mL), under a N_2_ atmosphere, water (2 mL) was added. The reaction was left overnight and confirmed to be complete by TLC. Upon completion, THF was removed in vacuo, and the resulting aqueous solution was acidified to pH 5 using 2 M HCl before each extraction into EtOAc (3 × 2 mL). The organic layers were combined before being washed with brine, dried over MgSO_4_, gravity-filtered, and concentrated in vacuo to leave the pure product as a white solid.

#### 4.1.4 General Method **3**

##### 4.1.4.1 The Reduction of Benzoates **2a** and **2b** to corresponding Benzyl Alcohols **4a** and **4b**

The corresponding methyl ester **2a** or **2b** was stirred in anhydrous DCM (2 mL) under a N_2_ atmosphere at −78 °C before 1 M DIBAL-H (1.5 mL) was added dropwise over 5 min. The reaction was allowed to warm R.T., and full conversion to the benzylic alcohols **4a** and **4b** was confirmed after 2 h by LCMS and TLC. The reaction mixture was subsequently quenched with water and filtered through Celite. The filtrate was concentrated in vacuo, and the resulting residue was purified by flash column chromatography, as described in each monograph.

#### 4.1.5 General Method **4**

##### 4.1.5.1 The Oxidation of Benzyl Alcohols **4a** and **4b** to corresponding Benzaldehydes **5a** and **5b**

The corresponding benzylic alcohol **4a** or **4b**, dissolved in the minimum amount of anhydrous DCM, was added dropwise to a mixture of DMP (1.1 equiv) in anhydrous DCM and stirred vigorously under an atmosphere of nitrogen overnight. The reaction was confirmed to be complete by TLC and subsequently diluted with DCM (5 mL) before being washed with NaHCO_3_/Na_2_S_2_O_3_ 1:1 (2 × 5.5 mL) and brine (5 mL). The organic layer was dried over MgSO_4_, gravity-filtered, and concentrated in vacuo to give the crude product that was purified by flash chromatography (eluent EtOAc/cyclohexane (cyHex) 10:90 to 60:40).

#### 4.1.6 General Method **5**

##### 4.1.6.1 Amide coupling

The relevant carboxylic acid (1.1 equiv) in a solution of DMF, DIPEA (1.5 equiv), and COMU (1.1 equiv) was stirred for 20 min at RT before the respective amine (1.0 equiv) was added. The reaction was confirmed to be complete via LCMS and TLC (eluent 1 M NH_3_ in MeOH/CHCl_3_ 10:90) before saturated NaHCO_3_/Water (1:1, 20 mL) was added and the mixture stirred for 20 min. The product was extracted into EtOAc (3 × 20 mL), and the organic layer was washed with water (60 mL) and brine (30 mL) before being dried over MgSO_4_, gravity-filtered, and concentrated under reduced pressure. The crude product was subsequently purified by flash column chromatography as described in each monograph.

#### 4.1.7 General Method **6**

##### 4.1.7.1 *N*-Boc-Deprotection

A solution of the relevant *N*-Boc-protected amine (1.0 equiv) dissolved in the minimum amount of 1,4-dioxane (0.1–1.5 mL) was stirred at 0 °C before an excess of 4 N HCl/1,4-dioxane (>20.0 equiv) was added. The reaction was stirred at RT until completion, which was confirmed by LCMS and TLC (eluent 1 M NH_3_ in MeOH/CHCl_3_ 10:90). Upon completion, the solvent was removed via concentration under reduced pressure to dryness to leave the corresponding amine as a HCl salt.

#### 4.1.8 General Method **7**

##### 4.1.8.1 Fluorescent Antagonist Generation

The HCl salt of the relevant amine **12a**–**h** (1.2 equiv) in DMF (1 mL) was treated with DIPEA (8.0 equiv) before being added to a solution of sulfo-Cy5-NHS (Lumiprobe) (1.0 equiv) in DMF (1 mL). The reaction was left overnight under the exclusion of light and confirmed to be complete by LCMS. Upon completion, the solvent was removed in vacuo, and the resulting residue was dissolved in water/acetonitrile 4:1 and purified by reverse-phase semipreparative high performance liquid chromatography (HPLC).

##### 4.1.8.2 Methyl 3-(2-((*tert*-Butoxycarbonyl)­amino) ethoxy)­benzoate (**2a**)

Methyl-3-hydroxybenzoate (**1b**) (1.0 g, 6.70 mmol) was functionalized following the Mitsunobu reaction described in **General Method 1**. The resulting crude residue (2.14 g) was purified using flash column chromatography (eluent Et_2_O/cyHex 10:90 to 80:20) to give the title compound (**2a**) (1.59 g 5.36 mmol, 81%). LCMS *m*/*z*: C_15_H_21_NO_5_ [MH]^+^ calcd 296.15 found: 296. *t*_R_ = 2.85 min (Method A). ^1^H NMR (400 MHz, CDCl_3_): δ 7.65 (d, *J* = 7.7 Hz, 1H), 7.55 (s, 1H), 7.35 (t, *J* = 8.0 Hz, 1H), 7.09 (dd, *J* = 8.3, 2.7 Hz, 1H), 4.98 (br s, 1H), 4.07 (t, *J* = 5.1 Hz, 2H), 3.91 (s, 3H), 3.55 (q, *J* = 5.5 Hz, 2H), 1.44 (s, 9H). ^13^C NMR (101 MHz, CDCl_3_): δ: 166.8, 158.6, 155.9, 131.5, 129.5, 122.4, 119.7, 114.8, 79.6, 67.4, 52.2, 40.1, 28.4.

##### 4.1.8.3 Methyl 4-(2-((*tert*-Butoxycarbonyl)­amino)­ethoxy)­benzoate (**2b**)

4-Hydroxybenzoate (**1b**) (1.08 g, 7.10 mmol) was functionalized following the Mitsunobu reaction described in **General Method 1**. The crude material (2.05 g) was purified using flash chromatography (eluent Et_2_O/cyHex 10:90 to 80:20) to give the title compound (**2b**) (1.46 g, 4.97 mmol, 70%). LCMS *m*/*z*: C_15_H_21_NO_5_ [MH]^+^:

296.15 found: 296.17 *t*_R_ = 2.82 min (Method A). ^1^H NMR (400 MHz, CDCl_3_): δ 7.99 (d, *J* = 8.7 Hz, 2H), 6.90 (d, *J* = 8.7 Hz, 2H), 4.97 (s, 1H), 4.07 (t, *J* = 5.1 Hz, 2H), 3.89 (s, 3H), 3.55 (q, *J* = 5.5 Hz, 2H), 1.45 (s, 9H). ^13^C NMR (101 MHz, CDCl_3_): δ: 166.8, 162.3, 155.9, 131.7, 123.0, 114.1, 79.7, 67.4, 51.9, 40.0, 28.4.

##### 4.1.8.4 3-(2-((*tert*-Butoxycarbonyl)­amino)­ethoxy)­benzoic Acid (**3a**)

Following **General Method 2,** methyl 3-(2-((*tert*-Butoxycarbonyl)­amino)­ethoxy)­benzoate (**2a**) (208 mg, 0.704 mmol) was hydrolyzed to the corresponding benzoic acid, leaving the title compound (**3a**) as a white crystalline solid (186 mg, 0.833 mmol, 95%). LCMS *m*/*z*: C_14_H_19_NO_5_ [MH]^+^ calcd 282.3 found: 282.2 *t*_R_ = 2.61 min (Method A). ^1^H NMR (400 MHz, DMSO): δ 12.96 (br s), 7.52 (d, *J* = 7.6, 1H), 7.47–7.37 (s, 1H), 7.24–7.10 (m, 1H), 7.00 (t, *J* = 5.8 Hz, 1H), 4.00 (t, *J* = 5.7 Hz, 2H), 3.30 (q, *J* = 5.8 Hz, 2H), 1.37 (s, 9H).^13^C NMR (101 MHz, DMSO): δ 167.1, 158.4, 155.7, 132.2, 129.7, 121.7, 119.2, 114.8, 77.8, 66.6, 28.2.

##### 4.1.8.5 4-(2-((*tert*-Butoxycarbonyl)­amino)­ethoxy)­benzoic Acid (**3b**)

Following **General Method 2,** methyl 4-(2-((*tert*-Butoxycarbonyl)­amino)­ethoxy)­benzoate (**2b**) (200 mg, 0.677 mmol) was hydrolyzed to the corresponding benzoic acid, leaving the title compound (**3b**) as a white solid (168 mg, 0.753 mmol, 89%). LCMS *m*/*z*: C_14_H_19_NO_5_ [MH]^+^ calcd 282.3 found: 282.2 *t*_R_ = 2.60 min (Method A). ^1^H NMR (400 MHz, DMSO): δ 12.6 (br s) 8.00–7.75 (d, *J* = 8.4 Hz, 2H), 7.00 (d, *J* = 8.4 Hz, 3H), 4.1 (br s, 1H) 4.07–3.94 (t, *J* = 5.7 Hz, 2H), 3.31 (q, *J* = 5.8 Hz, 2H), 1.37 (s, 9H). ^13^C NMR (101 MHz, DMSO): δ: 167.0, 162.1, 155.7, 131.4, 123.1, 114.3, 77.8, 66.7, 28.2.

##### 4.1.8.6 *tert*-Butyl (2-(3-(hydroxymethyl)­phenoxy)­ethyl)­carbamate (**4a**)

Following **General Method 3,** methyl 3-(2-((*tert*-Butoxycarbonyl)­amino)­ethoxy)­benzoate (**2a**) (205 mg, 0.694 mmol) was reduced and the resulting residue purified by flash column chromatography (eluent EtOAc/cyHex 10:90 to 50:50) to give the corresponding benzylic alcohol, title compound (**4a**) (165 mg, 0.617 mmol, 89%). LCMS *m*/*z*: C_14_H_21_NO_4_ [M + Na + H]^+^: calcd 290.1 found, 290.1 *t*_R_ = 2.80 min (Method A). ^1^H NMR (400 MHz, CDCl_3_): δ 7.3–7.2 (t, 1H), 7.0–6.9 (m, 2H), 6.8 (dd, *J* = 8.3, 2.6 Hz, 1H), 4.7–4.6 (br s, 1H), 4.0 (t, *J* = 5.1 Hz, 1H), 3.5 (q, *J* = 5.5 Hz, 1H), 1.5 (s, 9H).

##### 4.1.8.7 *tert*-Butyl (2-(4-(hydroxymethyl)­phenoxy)­ethyl)­carbamate (**4b**)

Following **General Method 3,** methyl 4-(2-((*tert*-Butoxycarbonyl)­amino)­ethoxy)­benzoate (**2b**) (198 mg, 0.670 mmol) was reduced and the resulting residue purified by flash column chromatography (eluent EtOAc/cyHex 10:90 to 50:50) to give the corresponding benzylic alcohol, title compound (**4b**) (151 mg, 0.565 mmol, 84%). LCMS *m*/*z*: C_14_H_21_NO_4_ [MH]^+^: calcd 290.1 found, 290.2 *t*_R_ = 2.79 min (Method A). ^1^H NMR (400 MHz, CDCl_3_): δ 7.9 (d, 2H), 6.9 (d, 2H), 5 (br s, 1H), 4.0 (t, *J* = 5.1 Hz, 1H), 3.5 (q, *J* = 5.5 Hz, 1H), 1.5 (s, 9H).

##### 4.1.8.8 *tert*-Butyl (2-(3-formylphenoxy)­ethyl)­carbamate (**5a**)

Following **General Method 4,** *tert*-Butyl (2-(3-(hydroxymethyl)­phenoxy)­ethyl)­carbamate (**4a**) (60 mg, 0.224 mmol) was oxidized and the resulting residue purified by flash column chromatography (eluent EtOAc/cyHex 10:90 to 60:40) to give the corresponding benzaldehyde, title compound (**5a**) (45 mg, 0.170 mmol, 76%). LCMS *m*/*z* C_14_H_19_NO_4_ -OMe [MH]^+^ calcd 296.2 found: 296.2 *t*_R_ = min (Method A). ^1^H NMR (400 MHz, CDCl_3_): δ 9.97 (s, 1H), 7.52–7.41 (m, 2H), 7.38 (dd, *J* = 2.8, 1.3 Hz, 1H), 7.17 (dt, *J* = 7.2, 2.3 Hz, 1H), 4.98 (br s, 1H), 4.08 (t, *J* = 5.2 Hz, 2H), 3.56 (q, *J* = 5.5 Hz, 2H), 1.45 (s, 9H). ^13^C NMR (101 MHz, CDCl_3_): δ: 190.8, 160.5, 155.8, 132.1, 130.3, 123.5, 120.2, 114.9, 79.8, 67.6, 40.0, 28.4.

##### 4.1.8.9 *tert*-Butyl (2-(4-formylphenoxy)­ethyl)­carbamate (**5b**)

Following **General Method 4,** *tert*-Butyl (2-(4-(hydroxymethyl)­phenoxy)­ethyl)­carbamate (**4b**) (60 mg, 0.224 mmol) was oxidized and the resulting residue purified by flash column chromatography (eluent EtOAc/cyHex 10:90 to 60:40) to give the corresponding benzaldehyde, title compound (**5b**) (33 mg, 0.125 mmol, 56%). LCMS *m*/*z* C_14_H_19_NO_4_–OMe exp 296.2 found 296.2 2 *t*_R_ = min (Method A). ^1^H NMR (400 MHz, CDCl_3_): δ 9.89 (s, 1H), 7.84 (dd, *J* = 8.5, 1.5 Hz, 2H), 7.00 (dd, *J* = 8.6, 1.5 Hz, 2H), 4.97 (t, *J* = 5.8 Hz, 1H), 4.11 (t, *J* = 5.2 Hz, 2H), 3.57 (q, *J* = 5.6 Hz, 2H), 1.45 (s, 9H). ^13^C NMR (101 MHz, CDCl_3_): δ: 190.8, 163.6, 155.8, 132.0, 130.3, 114.8, 79.8, 77.2, 67.6, 40.0, 28.4.

##### 4.1.8.10 *tert*-Butyl (3-(3*H*-spiro­[isobenzofuran-1,4′-piperidin]-1′-yl)­propyl)­carbamate (**7**)

To a solution of 3*H*-spiro­(isobenzofuran-1,4′-piperidine) hydrochloride (**6**) (1.860 g, 8.24 mmol, 1.0 equiv) in DMF (5 mL), *N*-Boc-3-bromo-propylamine (2.354 g, 9.88 mmol, 1.2 equiv) and K_2_CO_3_ (2.271 g, 16.48 mmol, 2.0 equiv) were added. The reaction mixture was heated at 60 °C and stirred overnight when it was confirmed complete by LCMS and TLC (eluent MeOH/DCM, 8:92). The solvent was then removed under reduced pressure, and the resulting oil was resuspended into EtOAc (50 mL) before being washed with aqueous saturated NH_4_Cl solution (2 × 50 mL) and brine (40 mL). The organic layer was dried over MgSO_4_, gravity-filtered, and concentrated under reduced pressure, giving the crude product (3.083 g, 0.465 mmol). Purification was carried out using flash column chromatography (eluent MeOH/DCM 1:99 to 8:92), to give the title compound (**7**) as a clear oil (2.40 g, 6.92 mmol, yield 84%). LCMS *m*/*z*: C_20_H_20_N_2_O_3_ [MH]^+^ calcd 347.2, found 347.1 *t*_R_ = 2.42 min (Method A). ^1^H NMR (400 MHz, CDCl_3_): δ 7.30–7.23 (m, 2H), 7.23–7.17 (m, 1H), 7.12 (m, 1H), 5.70 (br s, 1H), 5.07 (s, 2H), 3.23 (q, *J* = 6.2 Hz, 2H), 2.89 (d, *J* = 10.9, 2H), 2.51 (t, *J* = 6.8 Hz, 2H), 2.45–2.32 (m, 2H), 1.99 (td, *J* = 13.2, 4.5 Hz, 2H), 1.78 (m, 2H), 1.71 (p, *J* = 6.6 Hz, 2H), 1.45 (s, 9H). ^13^C NMR (101 MHz, CDCl_3_): δ: 156.2145.6, 138.9, 127.6, 127.4, 121.1, 120.8, 84.7, 78.8, 70.8, 57.2, 50.8, 50.2, 40.2, 36.7, 28.5, 26.4.

##### 4.1.8.11 3-(3*H*-Spiro­[isobenzofuran-1,4′-piperidin]-1′-yl)­propan-1-amine Dihydrochloride (**8**)

*Tert*-Butyl (3-(3*H*-spiro­[isobenzofuran-1,4′-piperidin]-1′-yl)­propyl)­carbamate (**7**) (422 mg, 1.22 mmol) in 1,4-dioxane (1 mL) was *N*-Boc-deprotected using 4 N HCl/1,4-dioxane (7.3 mL, 24 equiv) following **General Method 6,** leaving the title compound (**8**) as a white di-HCl salt (389 mg, 1.22 mmol) in quantitative yield. LCMS *m*/*z* C_15_H_22_N_2_O [MH]^+^: calcd 247.2 found 247.1 *t*_R_ = 0.54 min (Method A). ^1^H NMR (400 MHz, MeOD): δ 7.41–7.18 (m, 4H), 5.11 (s, 2H), 3.75–3.51 (m, 2H), 3.48–3.33 (m, 2H), 3.30–3.11 (m, 2H) 3.10 (t, *J* = 7.7 Hz, 2H), 2.43 (td, *J* = 14.2, 4.5 Hz, 2H), 2.32–2.10 (m, 2H), 2.10–1.84 (m, 2H).^13^C NMR (101 MHz, MeOD): δ 142.8, 138.7, 128.3, 127.5, 121.1, 120.3, 81.6, 70.9, 53.6, 49.8, 36.6, 33.5, 22.1.

##### 4.1.8.12 1-Benzyloxycarbonyl-*N*-[3-spiro­[isobenzofuran-1­(3*H*),4′-piperidine]-1-yl]­propyl-d-proline Amide (**9**)

Following **General Method 5**, Cbz-d-proline (335 mg, 1.35 mmol) was coupled to 3-(3*H*-spiro­[isobenzofuran-1,4′-piperidin]-1′-yl)­propan-1-amine (**8**) (389 mg, 0.122 mmol). The resulting crude residue (831 mg) was purified by silica gel column chromatography (eluent 1 M NH_3_ in MeOH/CHCl_3_ 1:99 to 10:90) to give the title compound (**9**) as a clear oil (552 mg, 1.16 mmol, 95%). LCMS *m*/*z*: C_28_H_35_N_3_O_4_ [MH]^+^ calcd 478.3 found, 478.9 *t*_R_ = 2.25 min (Method A). ^1^H NMR (400 MHz, DMSO): δ 8.0–7.9 (m, 1H), 7.4–7.2 (m, 9H), 5.1–5.0 (m, 2H), 5.0 (s, 2H), 4.1 (ddd, *J* = 19.7, 8.2, 2.9 Hz, 1H), 3.4 (m, 2H), 3.1 (m, 2H), 2.8–2.7 (m, 2H), 2.4–2.0 (m, 5H), 1.9–1.8 (m, 5H), 1.6–1.4 (m, 4H). ^13^C NMR (101 MHz, DMSO): δ 172.3, 172.1, 146.1, 139.3, 137.5, 128.9, 128.7, 128.2, 127.9, 127.7, 127.4, 121.7, 121.3, 84.4, 70.3, 66.2, 60.2, 50.2, 47.6, 37.6, 36.4, 31.8, 26.9, 24.4, 23.6.

##### 4.1.8.13 *N*-[3-Spiro­[isobenzofuran-1­(3*H*),4′-piperidine]-1-yl]­propyl-d-proline Amide (**10**)

Under an atmosphere of hydrogen, **9** (520 mg, 1.09 mmol) underwent hydrogenolysis in the presence of 10% Pd­(OH)_2_/C (60 mg) and methanol (20 mL). When the reaction was complete (4 h), the mixture was filtered through Celite and washed with methanol, and the filtrate was concentrated under reduced pressure. The resulting title compound (**10**) was isolated as a yellow oil (362 mg, 1.06 mmol) and was used without further purification. LCMS *m*/*z* C_20_H_29_N_3_O_2_ [MH]^+^: calcd 344.2 found 344.1 *t*_R_ = 0.37 min (Method A). ^1^H NMR (400 MHz, DMSO): δ 8.2 (t, *J* = 5.8 Hz, 1H), 7.3 (m, 4H), 5.0 (s, 2H), 3.6 (m, 1H), 3.1 (m, 2H), 2.9 (m, 2H), 2.8 (d, 2H), 2.4 (t, *J* = 6.9 Hz, 2H), 2.3–2.2 (m, 2H), 2.1–1.8 (m, 3H), 1.8–1.5 (m, 7H). ^13^C NMR (101 MHz, DMSO): δ: 172.5, 145.6, 138.8, 127.5, 127.2, 121.2, 120.8, 84.0, 69.8, 60.0, 56.1, 49.8, 48.6, 46.5, 37.3, 36.0, 30.3, 26.3, 25.2.

##### 4.1.8.14 *tert*-Butyl ((*S*)-1-((*R*)-2-((3-(3*H*-Spiro­[isobenzofuran-1,4′-piperidin]-1′-yl)­propyl)­carbamoyl)­pyrrolidin-1-yl)-1-oxopropan-2-yl)­carbamate (**11a**)

Following **General Method 5,** *N*-Boc-l-Ala-OH (24 mg, 0.128 mmol) was coupled to (**10**) (40 mg, 0.116 mmol) in DMF (1 mL). The reaction was confirmed complete by TLC and LCMS before purification was carried out via flash chromatography (eluent 1 M NH_3_ in MeOH/CHCl_3_ 1:99 to 10:90), leaving the title compound (**11a**) as a colorless oil (39 mg, 0.076 mmol, 65%). LCMS *m*/*z* C_28_H_42_N_4_O_5_ [MH]^+^: calcd 515.7 found: 515.2 *t*_R_ = 3.50 min (Method A). Analytical RP-HPLC *t*_R_ = 6.201 (Method B) purity >96%. ^1^H NMR (400 MHz, CDCl_3_): δ 7.34–7.23 (m, 1H), 7.23–7.16 (m, 2H), 7.16–7.07 (m, 1H), 5.26 (br s, 1H), 5.06 (s, 2H), 4.55 (d, *J* = 7.5 Hz, 1H), 4.37 (p, *J* = 6.9 Hz, 1H), 3.83 (t, *J* = 9.0 Hz, 1H), 3.50–3.41 (m, 1H), 3.41–3.30 (m, 1H) 3.26–3.13 (m, 1H), 2.86 (d, *J* = 11.2 Hz, 2H), 2.59–2.23 (m, 5H), 2.23–1.82 (m, 6H), 1.76 (d, *J* = 6.3 Hz, 4H), 1.43 (s, 9H), 1.31 (d, *J* = 1.3 Hz, 3H). ^13^C NMR (101 MHz, CDCl3): δ: 172.4, 170.7155.7, 145.7, 138.9, 127.6, 127.3, 121.1, 121.0, 120.8, 84.7, 80.0, 70.7, 60.5, 56.4, 50.2, 50.20, 48.3, 47.0, 38.2, 36.6, 24.5, 17.2. HRMS (ESI-TOF) *m*/*z*: calcd for C_28_H_42_N_4_O_5_ [M + H^+^], 515.3228; found, 515.3243.

##### 4.1.8.15 *tert*-Butyl (*R*)-(3-(2-((3-(3*H*-Spiro­[isobenzofuran-1,4′-piperidin]-1′-yl)­propyl)­carbamoyl)­pyrrolidin-1-yl)-3-oxopropyl)­carbamate (**11b**)

Following **General Method 5,** *N*-Boc-β-Ala-OH (27 mg, 0.142 mmol) was coupled to (**10**) (40 mg, 0.116 mmol) in DMF (1 mL). The reaction was confirmed complete by TLC and LCMS before purification was carried out via flash chromatography (eluent 1 M NH_3_ in MeOH/CHCl_3_ 1:99 to 10:90), leaving the title compound (**11b**) as a colorless oil (45 mg, 0.09 mmol, 75%). LCMS *m*/*z*: calcd for C_28_H_42_N_4_O_5_ [MH]^+^ calcd 515.7, found 515.2 *t*_R_ = 3.32 min (Method A). Analytical RP-HPLC *t*_R_ = 7.037 (Method B) purity >98%. ^1^H NMR (400 MHz, CDCl_3_): δ 7.35 (br s, 1H) 7.33–7.25 (m, 2H), 7.26–7.18 (m, 1H), 7.15 (m, 1H), 5.72 (br s, 1H), 5.09 (s, 2H), 4.53 (d, *J* = 8.0, 1H), 3.64–3.53 (m, 1H), 3.53–3.23 (m, 5H), 2.92 (br s, 2H), 2.72–2.31 (m, 6H), 2.31–1.86 (m, 5H), 1.77 (m, 5H), 1.45 (s, 9H). ^13^C NMR (101 MHz, CDCl_3_): δ 172.3, 171.5, 155.6, 145.9, 139.4, 128.1, 127.8, 121.5, 121.2, 85.0, 79.6, 77.7, 71.2, 60.4, 56.7, 50.8, 50.4, 47.9, 38.6, 37.0, 35.2, 30.2, 28.9, 28.9, 28.3, 25.4. HRMS (ESI-TOF) *m*/*z*: calculated for C_28_H_42_N_4_O_5_ [M + H^+^], 515.3228; found, 515.3243.

##### 4.1.8.16 *tert*-Butyl (*R*)-(4-(2-((3-(3*H*-Spiro­[isobenzofuran-1,4′-piperidin]-1′-yl)­propyl)­carbamoyl)­pyrrolidin-1-yl)-4-oxobutyl)­carbamate (**11c**)

Following **General Method 5,** *N*-Boc-GABA–OH (29 mg, 0.140 mmol) was coupled to (**10**) (40 mg, 0.116 mmol) in DMF (1 mL). The reaction was confirmed complete by TLC and LCMS before purification was carried out via flash chromatography (eluent 1 M NH_3_ in MeOH/CHCl_3_ 1:99 to 10:90) leaving the title compound (**11c**) as a colorless oil (47 mg, 0.09 mmol, 77%). LCMS *m*/*z*: C_29_H_44_N_4_O_5_ [MH]^+^ calcd 529.7; found 529.8 *t*_R_ = 3.42 min (Method A). Analytical RP-HPLC *t*_R_ = 7.003 (Method B) purity >99%. ^1^H NMR (400 MHz, CDCl_3_): δ 7.52 (br s, 1H), 7.25 (m, 2H), 7.23–7.16 (m, 1H), 7.13 (m, 1H), 5.06 (s, 2H), 4.84 (br s, 1H), 4.57–4.52 (m, 1H), 3.56 (t, *J* = 7.9 Hz, 1H), 3.45–3.20 (m, 4H), 3.12 (m, 1H), 2.87 (br s, 2H), 2.61–2.16 (m, 7H), 2.10–1.81 (m, 6H), 1.82–1.65 (m, 5H), 1.42 (s, 9H). ^13^C NMR (101 MHz, CDCl_3_): δ: 172.32, 171.36, 156.4, 145.6, 138.91, 127.59, 127.36, 121.05, 120.85, 84.7, 79.2, 70.7, 60.1, 50.2, 47.4, 39.6, 38.1, 36.6, 31.1, 29.7, 28.4, 25.3, 24.8, 22.9. HRMS (ESI-TOF) *m*/*z*: calcd for C_29_H_44_N_4_O_5_ [M + H^+^], 529.3385; found, 529.3404.

##### 4.1.8.17 *tert*-Butyl (*R*)-(5-(2-((3-(3*H*-Spiro­[isobenzofuran-1,4′-piperidin]-1′-yl)­propyl)­carbamoyl)­pyrrolidin-1-yl)-5-oxopentyl)­carbamate (**11d**)

Following **General Method 5,** *N*-Boc-5-Ava–OH (22 mg, 0.101 mmol) was coupled to (**10**) (30 mg, 0.087 mmol) in DMF (1.3 mL). The reaction was confirmed complete by TLC and LCMS before purification was carried out via flash chromatography (eluent 1 M NH_3_ in MeOH/CHCl_3_ 1:99 to 10:90) leaving the title compound (**11d**) as a colorless oil (22 mg, 0.04 mmol, 46%). LCMS *m*/*z*: C_30_H_46_N_4_O_5_ [MH]^+^ calcd 543.7; found, 543.8 *t*_R_ = 2.15 min (Method A). Analytical RP-HPLC *t*_R_ = 6.698 (Method B) purity > 99%. ^1^H NMR (400 MHz, CDCl_3_): δ 7.86 (br s, 1H), 7.34–7.15 (m, 4zzH), 5.07 (s, 2H), 4.86 (br s, 1H), 4.49–4.41 (m, 1H), 3.68 (ddd, *J* = 10.7, 7.5, 3.2 Hz, 1H), 3.49–3.26 (m, 5H), 3.18–2.80 (m, 5H), 2.50–2.37 (m, 3H), 2.36–2.13 (m, 3H), 2.12–1.86 (m, 4H), 1.83 (d, *J* = 14.2 Hz, 2H), 1.71–1.59 (p, *J* = 8.0 Hz, 2H), 1.52 (p, *J* = 7.3 Hz, 2fH), 1.40 (s, 9H). ^13^C NMR (101 MHz, CDCl3): δ: 172.9, 172.4, 156.1, 143.7, 138.4, 127.8, 127.7, 121.1, 121.1, 83.0, 71.2, 60.4, 54.3, 49.9, 49.0, 47.5, 40.2, 36.4, 34.2, 34.1, 29.6, 28.8, 28.4, 24.9, 23.9, 21.7. HRMS (ESI-TOF) *m*/*z*: calcd for C_30_H_46_N_4_O_5_ [M + H^+^], 543.3541; found, 543.3557.

##### 4.1.8.18 *tert*-Butyl (*R*)-(2-(3-(2-((3-(3*H*-Spiro­[isobenzofuran-1,4′-piperidin]-1′-yl)­propyl)­carbamoyl)­pyrrolidine-1-carbonyl)­phenoxy)­ethyl)­carbamate (**11e**)

Following **General Method 5,** 3-(2-((*tert*-Butoxycarbonyl)­amino)­ethoxy)­benzoic acid (**3a**) (22 mg, 0.08 mmol) was coupled to (**10**) (25 mg, 0.07 mmol) in DMF (1 mL). The reaction was confirmed complete by TLC and LCMS before purification was carried out via flash chromatography (eluent 1 M NH_3_ in MeOH/CHCl_3_ 1:99 to 10:90), leaving the title compound (**11e**) as a colorless oil (24 mg, 0.04 mmol, 55%). LCMS *m*/*z*: C_34_H_46_N_4_O_6_ [MH]^+^ calcd 607.3, found 607.8 *t*_R_ = 3.89 (Method A). Analytical RP-HPLC *t*_R_ = 6.630 (Method B) purity > 99%. ^1^H NMR (400 MHz, CDCl_3_): δ 7.46 (t, *J* = 5.6 Hz, 1H), 7.35–7.22 (m, 3H) 7.22–7.16 (m, 1H), 7.16–7.08 (m, 2H), 7.06 (s, 1H), 6.95 (dd, *J* = 8.4, 2.6 Hz, 1H), 5.05 (s, 2H), 5.01 (br s, 1H), 4.69 (dd, *J* = 7.6, 5.0 Hz, 1H), 4.06–3.92 (m, 2H) 3.64–3.43 (m, 3H), 3.43–3.27 (m, 2H), 2.88 (t, *J* = 11.5 Hz, 2H), 2.50 (t, *J* = 7.1 Hz, 2H), 2.47–2.34 (t,, *J* = 11.2 Hz, 3H), 2.23–1.92 (m, 5fH), 1.89–1.71 (m, 5H), 1.44 (s, 9H). ^13^C NMR (101 MHz, CDCl_3_): δ: 171.1, 170.6, 158.5, 155.9, 145.6, 138.9, 137.6, 129.6, 127.6, 127.3, 121.1, 120.8, 119.8, 116.5, 113.3, 84.6, 79.6, 70.7, 67.3, 60.1, 57.9, 56.5, 50.5, 50.3, 50.1, 40.0, 38.4, 36.5, 36.5, 30.9, 29.7, 28.4, 27.6, 26.3, 25.5, 22.7. HRMS (ESI-TOF) *m*/*z*: calcd for C_34_H_46_N_4_O_6_ [M + H^+^], 607.3490; found, 607.3514.

##### 4.1.8.19 *tert*-Butyl (*R*)-(2-(4-(2-((3-(3*H*-Spiro­[isobenzofuran-1,4′-piperidin]-1′-yl)­propyl)­carbamoyl)­pyrrolidine-1-carbonyl)­phenoxy)­ethyl)­carbamate (**11f**)

Following **General Method 5,** 4-(2-((*tert*-Butoxycarbonyl)­amino)­ethoxy)­benzoic acid (**3b**) (20 mg, 0.07 mmol) was coupled to (**10**) (26 mg, 0.08 mmol) in DMF (1 mL). The reaction was confirmed complete by TLC and LCMS before purification was carried out via flash chromatography (eluent 1 M NH_3_ in MeOH/CHCl_3_ 1:99 to 10:90), leaving the title compound (**11f**) as a colorless oil (40 mg, 0.07 mmol, 87%). LCMS *m*/*z*: C_34_H_46_N_4_O_6_ [MH]^+^ calcd 607.3, found 608.2 *t*_*R*_ = 3.88 (Method A). Analytical RP-HPLC *t*_R_ = 6.545 (Method B) purity > 98% ^1^H NMR (400 MHz, CDCl_3_): δ 7.53 (d, *J* = 8.3 Hz, 2H), 7.44 (s, 1H), 7.29–7.12 (m, 4H), 6.89 (d, *J* = 8.3 Hz, 2H), 5.05 (s, 2H), 5.01 (br s, 3H), 4.83–4.48 (m, 1H), 4.03 (t, *J* = 5.2 Hz, 2H), 3.74–3.59 (m, 1H), 3.53 (q, *J* = 5.6 Hz, 3H), 3.45–3.20 (m, 2H), 2.86 (d, *J* = 11.4 Hz, 2H), 2.50 (t, *J* = 6.9 Hz, 2H), 2.39 (t, *J* = 12.4 Hz, 2H), 2.17–1.86 (m, 6H), 1.84–1.71 (m, 4H), 1.45 (s, 9H). ^13^C NMR (101 MHz, CDCl_3_): δ: 171.4, 170.7, 160.3, 156.0, 145.7, 139.0, 129.6, 128.8, 127.7, 127.5, 121.2, 121.0, 114.2, 84.7, 79.8, 70.9, 67.4, 60.3, 56.5, 50.9, 50.4, 50.2, 40.1, 38.4, 36.6, 28.5, 27.7, 26.4, 25.8. HRMS (ESI-TOF) *m*/*z*: calcd for C_34_H_46_N_4_O_6_ [M + H^+^], 607.3490; found, 607.3512.

##### 4.1.8.20 *tert*-Butyl (*R*)-(2-(3-((2-((3-(3*H*-Spiro­[isobenzofuran-1,4′-piperidin]-1′-yl)­propyl)­carbamoyl)­pyrrolidin-1-yl)­methyl)­phenoxy)­ethyl)­carbamate (**11g**)

To a solution of *N*-[3-Spiro­[isobenzofuran-1­(3*H*),4′-piperidine]-1-yl]­propyl-d-proline amide (**10**) (39 mg, 0.113 mmol) and (**5a**) (33 mg, 0.124 mmol) in MeOH/AcOH 10:1 (1 mL), 2-picoline borane (14 mg, 0.124 mmol) was added and stirred under an atmosphere of nitrogen overnight. The reaction was confirmed complete by TLC (eluent 1 M NH_3_ in MeOH/CHCl_3_ 10:90) and LCMS before the solvent was removed in vacuo, and the resulting residue was resuspended in sodium carbonate Na_2_CO_3_ (10 mL). The product was extracted into EtOAc (3 × 10 mL) before being washed with brine (15 mL), dried over MgSO_4_, gravity-filtered, and concentrated under reduced pressure to leave the crude product (80 mg). Purification was carried out using flash chromatography (eluent 1 M NH_3_ in MeOH/CHCl_3_ 1:99 to 12:88), leaving the title compound (**11g**) as a colorless oil (45 mg, 0.076 mmol, 61%). LCMS *m*/*z*: C_34_H_48_N_4_O_5_ [MH]^+^ calcd 593.8 found, 593.7 *t*_R_ = 2.01 min (Method A). Analytical RP-HPLC *t*_R_ = 6.183 (Method B) purity >98%. ^1^H NMR (400 MHz, CDCl_3_): δ 7.57 (t, *J* = 6.1 Hz, 1H), 7.30–7.15 (m, 4H), 7.03 (d, *J* = 6.8 Hz, 1H), 6.90 (d, *J* = 7.5 Hz, 1H), 6.85 (s, 1H), 6.78 (dd, *J* = 8.2, 2.5 Hz, 1H), 5.23­(br s, 1H), 5.06 (s, 2H), 4.02 (t, *J* = 5.1 Hz, 2H), 3.84 (d, *J* = 13.1 Hz, 1H), 3.57–3.50 (q, *J* = 5.4 Hz, 2H), 3.50–3.43 (d, *J* = 13.0 Hz, 1H) 3.34 (p, *J* = 6.6 Hz, 1H), 3.26 (p, *J* = 6.6 Hz, 1H), 3.19 (dd, *J* = 10.0, 5.2 Hz, 1H), 3.08 (t, *J* = 7.7 Hz, 1H), 2.83 (br s, 2H), 2.55–2.30 (m, 4H), 2.23 (dq, *J* = 12.9, 9.1 Hz, 1H), 2.08–1.55 (m, 10H), 1.44 (s, 9H). ^13^C NMR (101 MHz, CDCl_3_): δ: 174.6, 158.9, 156.1, 140.5, 139.1, 129.7, 127.5, 121.6, 121.2, 120.9, 115.5, 112.8, 84.8, 79.7, 70.9, 67.8, 67.3, 60.0, 56.9, 54.3, 50.6, 50.3, 40.3, 37.8, 36.7, 30.9, 29.9, 28.6, 27.1, 24.2. HRMS (ESI-TOF): *m*/*z* calcd for C_34_H_48_N_4_O_5_ [M + H^+^], 593.3698; found, 593.3718.

##### 4.1.8.21 *tert*-Butyl (*R*)-(2-(4-((2-((3-(3*H*-Spiro­[isobenzofuran-1,4′-piperidin]-1′-yl)­propyl)­carbamoyl)­pyrrolidin-1-yl)­methyl)­phenoxy)­ethyl)­carbamate (**11h**)

To a solution of *N*-[3-Spiro­[isobenzofuran-1­(3*H*),4′-piperidine]-1-yl]­propyl-d-proline amide (**10**) (39 mg, 0.113 mmol) and (**5b**) (33 mg, 0.124 mmol) in MeOH/AcOH 10:1 (1 mL), 2-picoline borane (14 mg, 0.124 mmol) was added and stirred under an atmosphere of nitrogen overnight. The reaction was confirmed complete by TLC (eluent 1 M NH_3_ in MeOH/CHCl_3_ 10:90) and LCMS before the solvent was removed in vacuo, and the resulting residue was resuspended in sodium carbonate Na_2_CO_3_ (10 mL). The product was extracted into EtOAc (3 × 10 mL) before being washed with brine (15 mL), dried over MgSO_4_, gravity-filtered, and concentrated under reduced pressure to leave the crude product (82 mg). Purification was carried out using flash chromatography (eluent 1 M NH_3_ in MeOH/CHCl_3_ 1:99 to 12:88), leaving the title compound (**11h**) as a colorless oil (52 mg, 0.088 mmol, 71%). LCMS *m*/*z* calculated for C_34_H_48_N_4_O_5_ [MH]^+^:593.8, found 593.8 *t*_R_ = 2.01 min (Method A). Analytical RP-HPLC *t*_R_ = 6.183 (Method B) purity > 98%. ^1^H NMR (400 MHz, CDCl_3_): δ 7.61 (t, *J* = 5.7 Hz, 1H), 7.34–7.25 (m, 3H), 7.22 (d, *J* = 7.8 Hz, 2H), 7.06 (d, *J* = 6.4 Hz, 1H), 6.88 (d, *J* = 7.8 Hz, 2H), 5.09 (s, 2H), 5.04 (s, 1H), 4.02 (t, *J* = 5.1 Hz, 2H), 3.82 (d, *J* = 12.8 Hz, 1H), 3.54 (q, *J* = 5.5 Hz, 2H), 3.46 (d, *J* = 12.8 Hz, 1H), 3.39 (p, *J* = 6.6 Hz, 1H), 3.26 (p, *J* = 6.6 Hz, 1H), 3.19 (dd, *J* = 10.1, 5.2 Hz, 1H), 3.04 (t, *J* = 7.9 Hz, 1H), 2.87 (d, *J* = 9.1 Hz, 2H), 2.55–2.32 (m, 4H), 2.30–2.16 (m, 1H), 2.00 (m, 2H), 1.94–1.83 (m, 1H), 1.83–1.60 (m, 7H), 1.47 (s, 9H). ^13^C NMR (101 MHz, CDCl_3_): δ: 174.7, 158.1, 156.0, 139.1, 131.3, 130.2, 127.8, 127.5, 121.2, 120.9, 114.6, 79.7, 78.2, 70.9, 67.5, 67.4, 59.3, 56.9, 54.0, 50.6, 40.3, 37.8, 36.7, 30.9, 29.9, 28.5, 24.2. HRMS (ESI-TOF) *m*/*z*: calcd for C_34_H_48_N_4_O_5_ [M + H^+^], 593.3698; found, 593.3713.

##### 4.1.8.22 Potassium 1-(6-(((*S*)-1-((*R*)-2-((3-(3*H*-Spiro­[isobenzofuran-1,4′-piperidin]-1′-yl)­propyl)­carbamoyl)­pyrrolidin-1-yl)-1-oxopropan-2-yl)­amino)-6-oxohexyl)-3,3-dimethyl-2-((1*E*,3*E*)-5-((*E*)-1,3,3-trimethyl-5-sulfonatoindolin-2-ylidene)­penta-1,3-dien-1-yl)-3*H*-indol-1-ium-5-sulfonate (**13a**)

Following **General Method 6,** *tert*-Butyl ((*S*)-1-((*R*)-2-((3-(3*H*-spiro­[isobenzofuran-1,4′-piperidin]-1′-yl)­propyl)­carbamoyl)­pyrrolidin-1-yl)-1-oxopropan-2-yl)­carbamate (**11a**) (6.5 mg, 13 μmol) in 1,4-dioxane (1 mL) underwent *N*-Boc deprotection using 4 N HCl/1,4-dioxane (1 mL) to give (**12a**) (6.3 mg, 13 μmol) as a white 2.HCl salt in quantitative yield. LCMS *m*/*z*: C_23_H_34_N_4_O_3_[MH]^+^ calcd 415.5, found 415.5. The resulting intermediate (**12a**) (1.6 mg, 3.3 μmol) was functionalized with sulfo-Cy5-NHS (2.3 mg, 3.0 μmol) following **General Method 7**. Purification was carried out using RP-HPLC (eluent 0.1% HCOOH, MeCN/H_2_0 10:90 to 35:65) to give the title compound (**13a**) as a blue solid (3.1 mg, 2.9 μmol, 96%). LC-MS *m*/*z*: C_55_H_69_N_6_O_10_S_2_^–^ [MH]^+^ calcd 1039.3; found, 1038.9. HRMS (TOF ES^–^) [MH]^−^, calcd 1037.4522, found, 1037.4563 [M + Na + H]^+^ calcd 1061.4493; found, 1061.4526. Analytical RP-HPLC *t*_R_ = 4.609 min, purity > 99% (Method B).

##### 4.1.8.23 Potassium 1-(6-((3-((*R*)-2-((3-(3*H*-Spiro­[isobenzofuran-1,4′-piperidin]-1′-yl)­propyl)­carbamoyl)­pyrrolidin-1-yl)-3-oxopropyl)­amino)-6-oxohexyl)-3,3-dimethyl-2-((1*E*,3*E*)-5-((*E*)-1,3,3-trimethyl-5-sulfonatoindolin-2-ylidene)­penta-1,3-dien-1-yl)-3*H*-indol-1-ium-5-sulfonate (**13b**)

Following **General Method 6,** *tert*-Butyl (*R*)-(3-(2-((3-(3*H*-spiro­[isobenzofuran-1,4′-piperidin]-1′-yl)­propyl)­carbamoyl)­pyrrolidin-1-yl)-3-oxopropyl)­carbamate (**11b**) (2.8 mg, 5.4 μmol) underwent *N*-Boc deprotection using 4 N HCl/1,4-dioxane (1 mL) to give (**12b**) (1.9 mg, 3.9 μmol) as a white 2·HCl salt. LCMS *m*/*z* C_23_H_34_N_4_O_3_[MH]^+^: calcd: 415.5, found: 415.5. The resulting intermediate (**12b**) (1.9 mg, 3.9 μmol) was functionalized with sulfo-Cy5-NHS (2.3 mg, 3.0 μmol) following **General Method 7**. Purification was carried out using RP-HPLC (eluent 0.1% HCOOH, MeCN/H_2_0 10:90 to 30:70) to give the title compound (**13b**) as a blue solid (2.8 mg, 2.6 μmol, 91%). LCMS *m*/*z*: C_55_H_69_N_6_O_10_S_2_^–^ [MH]^+^: calcd 1039.31; found, 1038.9 *t*_R_ = HRMS (TOF ES^–^) [MH]^−^ calcd 1037.4522; found, 1037.4543 [M + Na + H]^+^ calcd 1061.4493; found, 1061.4532. Analytical RP-HPLC *t*_R_ = 4.576 min, purity > 99% (Method B).

##### 4.1.8.24 Potassium 1-(6-((4-((*R*)-2-((3-(3*H*-Spiro­[isobenzofuran-1,4′-piperidin]-1′-yl)­propyl)­carbamoyl)­pyrrolidin-1-yl)-4-oxobutyl)­amino)-6-oxohexyl)-3,3-dimethyl-2-((1*E*,3*E*)-5-((*E*)-1,3,3-trimethyl-5-sulfonatoindolin-2-ylidene)­penta-1,3-dien-1-yl)-3*H*-indol-1-ium-5-sulfonate (**13c**)

Following **General Method 6,** *tert*-Butyl (*R*)-(4-(2-((3-(3*H*-spiro­[isobenzofuran-1,4′-piperidin]-1′-yl)­propyl)­carbamoyl)­pyrrolidin-1-yl)-4-oxobutyl)­carbamate (**11c**) (9.1 mg, 17 μmol) underwent *N*-Boc deprotection using 4 N HCl/1,4-dioxane (1 mL) to give (**12c**) (8.2 mg, 16 μmol) as a 2.HCl salt in quantitative yield. LCMS *m*/*z*: C_24_H_36_N_4_O_3_[MH]^+^ 429.6, found 429.8. The resulting intermediate (**12c**) (1.6 mg, 3.27 μmol) was functionalized with sulfo-Cy5-NHS (2.3 mg, 3.0 μmol) following **General Method 7**. Purification was carried out using RP-HPLC (eluent 0.1% HCOOH, MeCN/H_2_0 10:90 to 30:70) to give the title compound (**13c**) as a blue solid (3.1 mg, 2.88 μmol, 96%). LCMS *m*/*z*: C_56_H_71_N_6_O_10_S_2_^–^ [MH]^+^ calcd 1053.3, found 1053.9 *t*_R_ = 4.21 (Method A) HRMS (TOF ES^–^) [MH]^−^: calcd 1051.4679, found, 1051.4725 [M + Na + H]^+^, calcd 1075.4644, found: 1075.4585. Analytical RP-HPLC *t*_R_ = 4.616 min, purity >99% (Method B).

##### 4.1.8.25 Potassium 1-(6-((5-((*R*)-2-((3-(3*H*-Spiro­[isobenzofuran-1,4′-piperidin]-1′-yl)­propyl)­carbamoyl)­pyrrolidin-1-yl)-5-oxopentyl)­amino)-6-oxohexyl)-3,3-dimethyl-2-((1*E*,3*E*)-5-((*E*)-1,3,3-trimethyl-5-sulfonatoindolin-2-ylidene)­penta-1,3-dien-1-yl)-3*H*-indol-1-ium-5-sulfonate (**13d**)

Following **General Method 6,** *tert*-Butyl (*R*)-(5-(2-((3-(3*H*-spiro­[isobenzofuran-1,4′-piperidin]-1′-yl)­propyl)­carbamoyl)­pyrrolidin-1-yl)-5-oxopentyl)­carbamate (**11d**) (8.0 mg, 14.7 μmol) underwent *N*-Boc deprotection using 4 N HCl/1,4-dioxane (1 mL) to give (**12d**) (7.7 mg, 14.9 μmmol) as a white 2·HCl salt in quantitative yield. LCMS *m*/*z*: C_25_H_38_N_4_O_3_[MH]^+^, 443.6; found, 443.6. The resulting intermediate (**12d**) (1.8 mg, 3.5 μmol) was functionalized with sulfo-Cy5-NHS (2.3 mg, 3.0 μmol) following **General Method 7**. Purification was carried out using RP-HPLC (eluent 0.1% HCOOH, MeCN/H_2_0 10:90 to 35:65) to give the title compound (**13d**) as a blue solid (3.2 mg, 3.0 μmol, 100%). LCMS *m*/*z*: C_57_H_73_N_6_O_10_S_2_^–^ [MH]^+^, calcd 1067.4; found, 1067.3 HRMS (TOF ES^–^): [MH]^−^ calcd 1065.4835; found, 1065.4934 [M + Na + H]^+^: calcd 1089.4800; found, 1089.4754. Analytical RP-HPLC *t*_R_ = 4.670 min, purity > 99% (Method B).

##### 4.1.8.26 Potassium 1-(6-((2-(3-((*R*)-2-((3-(3*H*-Spiro­[isobenzofuran-1,4′-piperidin]-1′-yl)­propyl)­carbamoyl)­pyrrolidine-1-carbonyl)­phenoxy)­ethyl)­amino)-6-oxohexyl)-3,3-dimethyl-2-((1*E*,3*E*)-5-((*E*)-1,3,3-trimethyl-5-sulfonatoindolin-2-ylidene)­penta-1,3-dien-1-yl)-3*H*-indol-1-ium-5-sulfonate (**13e**)

Following **General Method 6** *tert*-Butyl (*R*)-(2-(3-(2-((3-(3*H*-spiro­[isobenzofuran-1,4′-piperidin]-1′-yl)­propyl)­carbamoyl)­pyrrolidine-1-carbonyl)­phenoxy)­ethyl)­carbamate (**11e**) (11 mg, 18 μmol) in 1,4-dioxane (0.5 mL) underwent *N*-Boc deprotection using 4 N HCl/1,4-dioxane (0.5 mL) to give (**12e**) (10 mg, 18 μmol) as a 2HCl salt in quantitative yields. LCMS *m*/*z*: C_29_H_38_N_4_O_4_ [MH]^+^, 507.7; found, 507.7. The resulting intermediate (**12e**) (2.1 mg, 3.6 μmol) was functionalized with sulfo-Cy5-NHS (2.5 mg, 3.2 μmol) following **General Method 7**. Purification was carried out using RP-HPLC (eluent 0.1% HCOOH, MeCN/H_2_0 10:90 to 28:72) to give the title compound (**13e**) as a blue solid (2.2 mg, 1.9 μmol, 60%). LC-MS *m*/*z*: C_61_H_73_N_6_O_11_S_2_^–^ [MH]^+^: calcd 1131.4; found, 1131.3 *t*_R_ = 4.17 min (Method A) (HRMS (TOF ES^–^) [MH]^−^: calcd 1129.4784; found, 1129.4899 [M + Na + H]^+^: calcd 1153.4749; found, 1153.4679. Analytical RP-HPLC, purity >99% (Method B).

##### 4.1.8.27 Potassium 1-(6-((2-(4-((*R*)-2-((3-(3*H*-Spiro­[isobenzofuran-1,4′-piperidin]-1′-yl)­propyl)­carbamoyl)­pyrrolidine-1-carbonyl)­phenoxy)­ethyl)­amino)-6-oxohexyl)-3,3-dimethyl-2-((1*E*,3*E*)-5-((*E*)-1,3,3-trimethyl-5-sulfonatoindolin-2-ylidene)­penta-1,3-dien-1-yl)-3*H*-indol-1-ium-5-sulfonate (**13f**)

Following **General Method 6,** *tert*-Butyl (*R*)-(2-(4-(2-((3-(3*H*-spiro­[isobenzofuran-1,4′-piperidin]-1′-yl)­propyl)­carbamoyl)­pyrrolidine-1-carbonyl)­phenoxy)­ethyl)­carbamate (**11f**) (7 mg, 12 μmol) in 1,4-dioxane (0.5 mL) underwent *N*-Boc deprotection using 4 N HCl/1,4-dioxane (0.5 mL) to give (**12f**) (5.5 mg, 9.5 μmol) as a 2.HCl salt. LCMS *m*/*z* C_29_H_38_N_4_O_4_ [MH]^+^: calcd: 507.7 found: 507.7. The resulting intermediate (**12f**) (1.3 mg, 3.3 μmol) was functionalized with sulfo-Cy5-NHS (1.6 mg, 2.1 μmol) following **General Method 7**. Purification was carried out using RP-HPLC (eluent 0.1% HCOOH, MeCN/H_2_O 10:90 to 28:72) to give (**13f**) as a blue solid (2.3 mg, 1.9 μmol, 90%). LCMS *m*/*z* C_61_H_73_N_6_O_11_S_2_^–^ [MH]^+^: calcd 1131.4 found: 1131.3 *t*_R_ = 4.18 min (Method A) HRMS (TOF ES^–^) [MH]^−^: calcd 1129.4784 found: 1129.4899 [M + Na + H]^+^: calcd 1153.4749 found: 1153.4679. Analytical RP-HPLC, purity >99% (Method B).

##### 4.1.8.28 Potassium 1-(6-((2-(3-(((*R*)-2-((3-(3*H*-Spiro­[isobenzofuran-1,4′-piperidin]-1′-yl)­propyl)­carbamoyl)­pyrrolidin-1-yl)­methyl)­phenoxy)­ethyl)­amino)-6-oxohexyl)-3,3-dimethyl-2-((1*E*,3*E*)-5-((*E*)-1,3,3-trimethyl-5-sulfonatoindolin-2-ylidene)­penta-1,3-dien-1-yl)-3*H*-indol-1-ium-5-sulfonate (**13g**)

Following **General Method 6,** *tert*-Butyl (*R*)-(2-(3-((2-((3-(3*H*-spiro­[isobenzofuran-1,4′-piperidin]-1′-yl)­propyl)­carbamoyl)­pyrrolidin-1-yl)­methyl)­phenoxy)­ethyl)­carbamate (**11g**) (10 mg, 17 μmol) underwent *N*-Boc deprotection using 4 N HCl/1,4-dioxane (1 mL) to give (**12g**) (9.4 mg, 17 μmol) as a white 3.HCl salt in quantitative yields. LCMS *m*/*z* C_29_H_40_N_4_O_3_ [MH]^+^: 493.7, found 493.6. The resulting intermediate (**12g**) (2.6 mg, 4.3 μmol) was functionalized with sulfo-Cy5-NHS (2.3 mg, 3.0 μmol) following **General Method 7**. Purification was carried out using RP-HPLC (eluent 0.1% HCOOH, MeCN/H_2_0 10:90 to 28:72) to give the title compound (**13g**) as a blue solid (3.2 mg, 2.8 μmol, 94%). LCMS *m*/*z*: C_61_H_75_N_6_O_10_S_2_^–^ [MH]^+^ calcd 1117.4; found, 1118.5 *t*_R_ = 3.72 min (Method A) HRMS (TOF ES^–^): [MH]^−^ calcd 1115.4992; found, 1115.5098 [M + Na + H]^+^: calcd 1139.4962; found, 1139.4863. Analytical RP-HPLC *t*_R_ = 5.481 min, purity > 99% (Method B) Supplementary Figure S4.

##### 4.1.8.29 Potassium 1-(6-((2-(4-(((*R*)-2-((3-(3*H*-Spiro­[isobenzofuran-1,4′-piperidin]-1′-yl)­propyl)­carbamoyl)­pyrrolidin-1-yl)­methyl)­phenoxy)­ethyl)­amino)-6-oxohexyl)-3,3-dimethyl-2-((1*E*,3*E*)-5-((*E*)-1,3,3-trimethyl-5-sulfonatoindolin-2-ylidene)­penta-1,3-dien-1-yl)-3*H*-indol-1-ium-5-sulfonate (**13h**)

Following **General Method 6,** *tert*-Butyl (*R*)-(2-(4-((2-((3-(3*H*-spiro­[isobenzofuran-1,4′-piperidin]-1′-yl)­propyl)­carbamoyl)­pyrrolidin-1-yl)­methyl)­phenoxy)­ethyl)­carbamate (**11h**) (8 mg, 14 μmol) underwent *N*-Boc deprotection using 4 N HCl/1,4-dioxane (1 mL, 4 mmol, 286 equiv) to give (**12h**) (8.5 mg, 14 μmol) as a 3.HCl salt in quantitative yields. LCMS *m*/*z*: C_29_H_40_N_4_O_3_ [MH]^+^, 493.7; found, 493.6. The resulting intermediate (**12h**) (1.2 mg, 2.0 μmol) was functionalized with sulfo-Cy5-NHS (1.41 mg, 1.8 μmol) following **General Method 3**. Purification was carried out using RP-HPLC (eluent 0.1% HCOOH, MeCN/H_2_0 10:90 to 28:72) to give the title compound (**13h**) as a blue solid (1.8 mg, 1.6 μmol, 86%). LCMS *m*/*z*: C_61_H_75_N_6_O_10_S_2_^–^ [MH]^+^, calcd 1117.4; found, 1118.6 *t*_R_ = 2.7 (Method A). HRMS (TOF ES^–^): [MH]^−^ calcd 1115.4992, found, 1115.5098 [M + Na + H]^+^, calcd 1139.4962; found, 1139.4863. Analytical RP-HPLC *t*_R_ = 5.429 min, purity >99% (Method B) Supporting Figure S4.

##### 4.1.8.30 (*R*,*E*)-*N*-(3-(3*H*-Spiro­[isobenzofuran-1,4′-piperidin]-1′-yl)­propyl)-1-(4-(2-(6-(2-(4-(2-(5,5-Difluoro-7-(thiophen-2-yl)-5*H*-4λ^4^,5λ^4^-dipyrrolo­[1,2-*c*:2′,1′-*f*]­[1,3,2]­diazaborinin-3-yl)­vinyl)­phenoxy)­acetamido)­hexanamido)­ethoxy) benzyl)­pyrrolidine-2-carboxamide (**14h**)

**12h** (2.1 mg, 3.5 μmol) in DMF (1 mL) and DIPEA (4 μL, 20 μmol) was added to BODIPY-630/650-X-NHS (Lumiprobe) (1.9 mg, 2.9 μmol) in DMF (1 mL). The reaction was left overnight under the exclusion of light and confirmed to be complete by LCMS before the DMF was removed under reduced pressure. The resulting solid was redissolved in MeCN/H_2_0 (1 mL, 2:3) and purified using semipreparative RP-HPLC (eluent 0.1% HCOOH, MeCN/H_2_0 25:75 to 50:50) to give the title compound (**14h**) as a blue solid (2.44 mg, 2.4 μmol, 81%). LCMS *m*/*z*: C_58_H_66_BF_2_N_7_O_6_S [MH]^+^ calcd 1038.5 found: 1038.6 *t*_R_ = 2.7 (Method A). HRMS (TOF ES^–^) [M + H]^+^: calcd: 1038.4856 found: 1038.4963. Analytical RP-HPLC *t*_R_ = 6.383 min, purity > 99% (Method B).

### 4.2 Pharmacology Experiment

#### 4.2.1 SNAP-Tagged Human NOPr Flp-In CHO Stable Cell Line Generation

Flp-In Chinese hamster ovary (CHO) cells were grown in Dulbecco’s modified Eagle’s medium (DMEM) (Sigma-Aldrich, D6421) supplemented with 10% v/v fetal bovine serum (FBS), l-glutamine (2 mM), and the antibiotic Zeocin (1 μg·mL^–1^). Using a 6:1 ratio of the transfection reagent polyethylenimine/DNA, the FlpIn CHO cells were transfected with the following plasmids: pOG44, the Flp-recombinase expression vector, and a pcDNA5 vector carrying the full sequence of the SNAP-NOPr. Cells were collected 24 h post-transfection and reseeded into new flasks with Media containing the antibiotic hygromycin (600 μg·mL^–1^) as a selection agent. After 3 weeks under antibiotic pressure, cells were sorted using fluorescence-activated cell sorting (FACS), as shown in Supporting Figure S5, and experiments were carried out using cells grown from single cell clones stably expressing high levels of NOPr.

#### 4.2.2 Cell Culture

Flp-In CHO cells stably expressing SNAP-NOPr were maintained in Dulbecco’s modified Eagle medium (DMEM) (Sigma-Aldrich, D6421) supplemented with 10% *v*/*v* Fetal Bovine Serum (FBS) (Sigma-Aldrich), l-glutamine (2 mM), and hygromycin (200 μg·mL^–1^). HEK 293T cells were maintained in DMEM (Sigma-Aldrich, D5796) supplemented with 10% *v/v* FBS (Sigma-Aldrich).

All cell lines were incubated at 37 °C in a humidified atmosphere of 95% air and 5% CO_2_. Cells were passaged using 0.05% Trypsin–EDTA (Thermo Fisher Scientific, Loughborough, UK), centrifuged at 1000 rpm for 3 min, and counted using a Countess II Automated Cell Counter (Thermo Fisher Scientific).

#### 4.2.3 BRET GPA Assay

##### 4.2.3.1 Cell Seeding

Media was aspirated, and cells were rinsed with PBS (5 mL). Cells were then detached using 0.05% Typsin-EDTA and plated into 10 cm culture dishes (∼7 × 10^5^ cells for the next day transfection and ∼ 3.5 × 10^5^ cells for the day after).

##### 4.2.3.2 BRET Assay Transfection

Flp-In CHO cells stably expressing SNAP-NOPr were transiently transfected with Gα_i2_ (2 μg), Gβ_1_-Venus (156–259)­(1 μg), Gγ_2_-Venus­(1–155)­(1 μg), and masGRK3ct-RLuc8 (1 μg) using polyethylenimine (PEI) in a 1:6 DNA/PEI ratio. The mixture was suspended in sterile NaCl, vortexed, and then incubated (10 min, RT), before being added to the cells in fresh antibiotic media DMEM (7 mL) supplemented with 10% FBS (*v/v*) and penicillin (50 units·mL^–1^)- streptomycin (50 μg.mL^–1^).

##### 4.2.3.3 Cell Plating

Antibiotic media was aspirated, and then cells were detached using 0.05% Typsin-EDTA, resuspended in media, DMEM supplemented with 10% FBS (11 mL), and replated (100 μL/well) into white sterile 96-well CulturPlates. Cells were then incubated overnight (37 °C, 5% CO_2_ humidified atmosphere).

##### 4.2.3.4 BRET Assay

Media was aspirated before cells were washed with PBS (80 μL/well) and then incubated in PBS (70 μL/well for antagonist mode or 80 μL/well for agonist mode) for 30 min at 37 °C in a humidified atmosphere of 95% air and 5% CO_2_. Cells were then incubated with coelenterazine H (NanoLight Technology) (10 μL/well, 50 μM, 5 min, RT) before ligands were added to the 96-well plate with final ligand concentrations ranging from (10 μM-0.01 nM). In **Antagonist Mode,** an EC_80_ concentration of N/OFQ (10 μL/well) and competitors **11a-h, 13g-h,** and **14h** (10 μL/well) were added, and in **Agonist Mode,** N/OFQ (10 μL/well) or **11a-h, 13g-h,** and **14h** (10 μL/well) was added. Cells were incubated in the plate reader, and luminescence was read using dual emission detection at 475 nm (Rluc8 Donor) and 535 nm emission (BRET signal) at 10 and 30 min. Plates were read on a PHERAstar (BMG Lab Tech), and data were exported through MARS and analyzed using Prism (GraphPad, Version 10). The data were normalized to the average maximum N/OFQ response and baseline buffer before being fit to three-parameter eq 1 to derive values of agonist potency (EC_50_, EC_80_) or inhibitory potency (IC_50_).

1 $$Y=Bottom+\frac{\left(Top-Bottom\right)}{1+10^{\log{EC}_{50}-X}}$$

Where *Y* = the response expressed as a percentage of N/OFQ maximal response, and *X* is the logarithm of ligand concentration.

#### 4.2.4 Confocal Microscopy

For all experiments, live cell single time point confocal images were captured using a Zeiss Cell Discoverer 7 LSM 900 high-content automated confocal microscope. Two images per well were captured using a 10× objective and the Cy5 channel (650 λ_ex_, 673 λ_em_), the AF488 (495λ_ex_, 520 λ_em_) channel, and the CFP (433λ_ex_, 475 λ_em_) channel. All cells were imaged in an imaging buffer containing (2 mM sodium pyruvate, 145 mM NaCl, 10 mM d-glucose, 5 mM KCl, 1 mM MgSO_4_·7H_2_O, 10 mM HEPES, 1.3 mM CaCl_2_ dihydrate, and 1.5 mM NaHCO_3_).

##### 4.2.4.1 Initial Screen Experiment

Stably expressing SNAP-NOPr FlpIn-CHO cells suspended in DMEM (D6421) (100 μL) were seeded (10,000 cells/well) into 96-well poly-d-lysine (PDL)-coated Greiner black polyester plates. Cells were incubated for 48 h at 37 °C and 5% CO_2_ prior to imaging.

Before measurement, the media were removed, and cells were washed with 90 μL of imaging buffer. Cells were then incubated with SNAP-AF488 (New England Biolabs) (0.5 μg·mL^–1^) in imaging buffer for 30 min at 37 °C. After incubation, the SNAP-AF488 solution was aspirated and cells were rinsed twice with imaging buffer (90 μL). Fluorescent C24-based ligands **13a**-**h** and **14h** (1 μM) in imaging buffer (100 μL) were added in the presence and absence of SB-612111­(1 μM). Cells were incubated for 30 min at 37 °C and 5% CO_2_ before the images were captured.

##### 4.2.4.2 Imaging Selectivity Experiment

HEK 293T cells suspended in DMEM (Sigma-Aldrich, D5796) and 10% *v/v* FBS (Sigma-Aldrich) were seeded (20,000 cells/well) into 96-well PDL-coated Greiner black polyester plates. 24 h after plating, cells were transiently transfected with SNAP-humanNOPr; SNAP hMOPr; SNAP hDOPr or SNAP hKOPr (100 ng/well) and NLS-CFP (50 ng/well) using FuGENE HD (Promega, UK) in a 3:1 ratio (FuGENE/DNA). The mixture was suspended in Opti-MEM, vortexed, and incubated for 10 min before being added to the cells in fresh antibiotic media (100 μL). Cells were imaged 48 h post-transfection following the same protocol as “initial screen”, but cells were incubated with fluorescent ligands **13g** (400 nM (∼20-fold [*K*_D_])) and **13h** (100 nM (∼20-fold [*K*_D_])). All images were acquired using the same laser and optical settings.

##### 4.2.4.3 Image Analysis

All images were exported as .czi files using ZEN (blue addition) and analyzed in Fiji (ImageJ). For selectivity experiments, cells expressing NLS-CFP were counted automatically in Fiji. Images were thresholded and converted to binary, and clustered cells were separated using the watershed function. The number of cells was then determined using the “analyze particles” tool. The total fluorescence intensity from the Cy5 channel was normalized to the number of cells in each corresponding image to calculate fluorescence intensity/cell. The mean ± SEM from *n* = 3 experiments performed in duplicate was used to generate bar graphs in GraphPad Prism (Version 10) and statistical analysis was performed using repeated measures one-way ANOVA with Dunnet’s multiple comparisons. This confirmed significant selectivity of **13g**-**h** for the NOPr (*p* < 0.05).

#### 4.2.5 Terbium Labeling and Membrane Preparation for TR-FRET-Based Assays

##### 4.2.5.1 SNAP-Lumi4-Tebium Cryptate Labeling and Membrane Preparation

Flp-In-CHO cells stably expressing SNAP-humanNOPr and Flp-In CHO cells transiently transfected with SNAP-hMOPr, SNAP-hDOPr, or SNAP-hKOPr (15.5 μg/T175) using PEI in a 1:6 DNA/PEI ratio were grown to approximately full confluency in T175 flasks. Media was aspirated before cells were washed with PBS (2 × 12 mL) and then incubated with SNAP-Lumi4-Tb in 1× SNAP-CLIP labeling reagent (1 h, 37 °C, 5% CO_2_ humidified atmosphere). The labeling reagent was then removed, and the cells washed with PBS (12 mL) before being harvested in PBS (12 mL) by scraping. Cells were then centrifuged (1500 rpm, 10 min), the supernatant was removed, and the cell pellet was frozen (−80 °C).

Membranes were prepared by thawing the cell pellets on ice before suspension in cold PBS (15 mL). The solution was homogenized using a hand-held homogenizer (T10 basic ULTRA-TURRAX, IKA) (10 × 2 s) before being centrifuged (Beckman Avanti J-25, 48,000*g*, 30 min, 4 °C). The supernatant was removed, and the cell pellet was resuspended in cold PBS (15 mL) and centrifuged again. After the supernatant was discarded, the cell pellet was resuspended in PBS (4 mL) and homogenized using an overhead stirrer (RW 16 basic, IKA).

Membrane protein concentration was measured using bicinchoninic acid (BCA, Thermo Fisher Scientific) assay with absorbance measured at 570 nm on a PHERAstar plate reader (BMG Labtech). A standard curve was generated using increasing concentrations of bovine serum albumin (BSA) and analyzed in Microsoft Excel version 16.99.1 to quantify membrane protein concentration. All samples were measured in duplicate, and the mean value was used for quantification. The cell membrane solution was subsequently aliquoted and stored (−80 °C).

#### 4.2.6 TR-FRET Assays

##### 4.2.6.1 TR-FRET Binding and Kinetic Assays

All ligands were prepared in TR-FRET buffer, consisting of Hanks’ Balanced Salt Solution (Sigma-Aldrich H6648) (KCl 5 mM, KH_2_PO_4_ 0.4 mM, NaHCO_3_ 4 mM, NaCl 137 mM, Na_2_HPO_4_ 0.3 mM), Pluronic-F127 (0.02% w/v), DMSO (1% v/v), and glucose (1 mg.mL^–1^), adjusted to pH 7.4. Membrane aliquots were thawed on ice prior to use, and membrane suspensions [membrane (0.05 μg.μL^–1^), Gpp­(NH)p (100 μM), and saponin (50 μg.mL^–1^)] were prepared in TR-FRET buffer. To ensure homogeneity, the membrane suspension was passed through a 27-gauge needle before use. Measurements were made in low-binding 384-well optiplates (PerkinElmer) on a PHERAstar (BMG Labtech) plate reader using the HTRF module [337 nm excitation with dual emission detection at 620 nm (terbium cryptate donor) and 665 nm emission (fluorescent ligand acceptor)] at a focal height of 11.4 mm. Integration was started 60 μs post irradiation (4 flashes/well) for 400 μs, all saturation and competition binding experiments were carried out in duplicate wells, and data were reported as the mean HTRF ratio $\left(\left(\frac{acceptor\;emission}{donor\;emission}\right)\times 10,000\right)$ from 3 repeat measurements ±SEM. Kinetic binding experiments were performed in singlet wells, and the data represent the mean of *n* = 3 experiments ±SEM.

##### 4.2.6.2 Equilibrium Saturation Binding Experiments

Increasing concentrations of fluorescent ligand (FL) (**13g**-**h**) or opioid red antagonist (Cisbio) (10 μL) were added to either TR-FRET buffer (10 μL) or a competing antagonist solution [SB-612111 for NOPr and Naloxone for MOPr, DOPr, and KOPr (10 μL, 10 μM final concentration)] to measure the total and nonspecific binding, respectively. Membrane solution (20 μL) was added, and the measurement was taken after a 2 h incubation at 37 °C. All data were converted to specific binding (total – nonspecific) and fitted using eq 2 to determine *K*_D_.

2 $$HTRF\;ratio\times 10,000=B_{\max}\frac{\left[FL\right]}{\left(K_{D}+\left[FL\right]\right)}$$

##### 4.2.6.3 Kinetic Binding Experiments

Membrane suspension was primed into the PHERAstar injectors before being injected (20 μL, 400 μL/s) into wells containing increasing concentrations of fluorescent ligands **13g**-**h** (10 μL) and TR-FRET buffer (10 μL) (total binding) or SB-612111 (10 μL, 10 μM) (nonspecific binding). Measurement was taken every 4 s over 15 min at room temperature. Specific binding curves (total – nonspecific binding) were plotted for each concentration ([L]) and globally fit using Prism (GraphPad, Version 10) with the “association kinetics (two or more concentrations)” model to derive the association rate (*K*_on_) and the dissociation rate (*K*_off_), as shown in eq 3.

3 $$K_{obs}=\left[L\right]\times K_{on}+K_{off}$$

##### 4.2.6.4 Competition Binding Experiments

Fluorescent ligands **13g** (20 nM) or **13h** (5 nM) at an NOPr-*K*_D_ concentration were used as fluorescent tracers in TR-FRET binding experiments. Fluorescent ligands (10 μL) were added to increasing concentrations of the competing ligand (10 μL). Membrane solution (20 μL) was added, and the measurement was taken after a 2 h incubation at 37 °C. Competition binding curves were plotted for each ligand and fitted using Prism (GraphPad, Version 10) with the “one site -fit *K*_i_” model, where IC_50_ values are converted to *K*_i_ values via the Cheng–Prusoff eq 4 using the *K*_D_ of the fluorescent ligand (FL).

4 $$K_{i}=\frac{\left[{IC}_{50}\right]}{\left(1+\frac{\left[FL\right]}{K_{D}}\right)}$$

##### 4.2.6.5 Saturation and Competition Binding Experiments in the Presence and Absence of Sodium and Gpp­(NH)­p

Saturation and competition binding experiments were performed as previously described using different buffer conditions. In the sodium and Gpp­(NH)­p, free condition experiments were carried out in buffer containing (20 mM HEPES, 100 mM NMDG, 6 mM MgCl_2_, 1 mM EGTA, and 1 mM EDTA, pH 7.4) and in the sodium and Gpp­(NH)­p containing condition buffer containing (20 mM HEPES, 100 mM NaCl, 6 mM MgCl_2_, 1 mM EGTA, and 1 mM EDTA, pH 7.4) with 100 μM Gpp­(NH)­p. In competition binding experiments, membranes (Lumi4-Tb-SNAP-NOR-CHO) (20 μL) were incubated with 10 μL fluorescent ligand **13h** (10 nM) and increasing concentrations of competing ligands **MCOPPB** (10^–6^ to 10^–1**2.5**^ M), **SB-612111** (10^–5^ to 10^–1**1.5**^ M), or **N**/**OFQ** (10^–5^ to 10^–1**2.5**^ M). For saturation experiments, increasing concentrations of **13h** (0–400 nM) were used.

## Supplementary Material





## Notes

Molecular formula strings of tested compounds (CSV)
Single time point confocal images of **13a**-**h** and **14h** applied to live FlpIn CHO cells stably expressing SNAP-NOPr; TR-FRET equilibrium saturation binding curves for **13g** and **13h** to Lumi4-Tb SNAP-NOPr, Lumi4-Tb SNAP-MOPr, Lumi4-Tb SNAP-DOPr and Lumi4-Tb SNAP-KOPr in CHO-membranes; TR-FRET saturation binding curves and determined affinity data for **13h** to Lumi4-Tb SNAP-NOPr CHO membranes in buffers containing 100 mM Na^+^ and 100 μM Gpp­(NH)­p, or 100 mM NMDG; HPLC chromatograms for **13g** and **13h**; and FACS data for the generation of Flp-In CHO cells stably expressing SNAP-NOPr (PDF)

## Abbreviations USED

AF488: Alexafluor488
BRET: bioluminescence resonance energy transfer
BODIPY-X 630/650: 6-(((4,4-difluoro-5-(2-thien-yl)-4-bora-3*a*,4*a*-diaza-*s*-indacene-3-yl)­styryloxy) acetyl)­aminohexanoic acid
Boc: *tert*-Butyloxycarbonyl
Cbz: carboxybenzyl
cyHex: cyclohexane
CHO: Chinese hamster ovary
CI: confidence interval
COMU: 1-cyano-2-ethoxy-2-oxoethylidenaminooxy)­dimethylamino-morpholino-carbenium hexafluorophosphate
DCM: dichloromethane
DIBAL-H: diisobutylaluminum hydride
DIAD: diisopropyl azodicarboxylate
DIPEA: diisopropylethylamine
DMP: Dess-Martin periodinane
DOPr: δ opioid receptor
ESI: electron spray ionization
GPCR: G-protein coupled receptor
FACS: Fluorescence-activated cell sorting
GPA: G-protein activation
GRK: G-protein coupled receptor kinase
HEK: human embryonic kidney
HRMS: high resolution mass spectrometry
HTRF: homogeneous TR-FRET
KOR: κ opioid receptor
LCMS: liquid chromatography mass spectrometry
MOPr: μ opioid receptor
N/OFQ: nociceptin/orphanin FQ
NOPr: nociceptin opioid peptide receptor
NSB: nonspecific binding
OR: opioid receptor
RP-HPLC: reverse phase high performance liquid chromatography
SAR: structure–activity relationship
Sulfo-Cy5-NHS: 1-[6-(2,5-dioxopyrrolidin-1-yl)­oxy-6-oxohexyl]-3,3-dimethyl-2-[5-(1,3,3-trimethyl-5-sulfonatoindol-1-ium-2-yl)­penta-2,4-dienylidene]­indole-5-sulfonate
THF: tetrahydrofuran
TLC: thin layer chromatography
TR-FRET: time-resolved fluorescence resonance energy transfer
TPPO: triphenylphosphine oxide
