# Supplementary material for: Design, Synthesis, and Characterization of Novel, Subtype-Selective Fluorescent Antagonists Targeting the Nociceptin/Orphanin FQ Opioid Peptide Receptor
Source: J Med Chem. 2025 Dec 31;69(2):1322–41. doi: 10.1021/acs.jmedchem.5c02707 (PMC12833874; doi:10.1021/acs.jmedchem.5c02707)
Supplement: Supplementary file 2 [file jm5c02707_si_002.pdf]

Supporting Information

Design, synthesis and characterization of novel, subtype selective fluorescent antagonists targeting the nociceptin/orphanin FQ opioid peptide receptor

George J Farmer[1,2,3] , Julie Sanchez[2,3], Annabell Millns[1], Tamzin Antony[2], Meritxell Canals[1,3], J. Robert Lane[1,3,*] , Shailesh N. Mistry[2,3,*].

[1] Division of Physiology, Pharmacology and Neuroscience, Medical School, School of Life Sciences, University of Nottingham, Nottingham NG7 2UH, UK

[2] Division of Biomolecular Science and Medicinal Chemistry, School of Pharmacy, University of Nottingham Biodiscovery Institute, University Park, University of Nottingham, Nottingham, NG7 2RD, UK

[3] Centre of Membrane Proteins and Receptors, School of Life Sciences, University of Nottingham, Medical School, Queens Medical Centre, Nottingham, NG7 2UH, UK

Corresponding Authors
*J. Robert Lane - Phone:+44-115-8230468 ; Email: rob.lane@nottingham.ac.uk

*Shailesh N. Mistry - Phone: +44-115-8467983; Email: Shailesh.mistry@nottingham.ac.uk

Table of contents

		Page
Table S1	Structure and opioid receptor affinity of selective NOPr antagonist SB612111	S2
Figure S1	Single time point confocal images of live FlpIn CHO cells stably expressing SNAP-NOPr	S3
Figure S2	TR-FRET binding experiments to characterise the affinity of fluorescent ligand **13g** and **13h** to the NOPr	S4
Figure S3	Figure S3. TR-FRET equilibrium saturation binding experiments to determine the selectivity of fluorescent ligands **13g-h** against the NOPr; MOPr; DOPr and KOPr	S5
Figure S4	TR-FRET saturation binding experiments to characterize the affinity of fluorescent ligand **13h** in buffers containing a) 100 mM Na^+ and 100 μM Gpp(NH)p or b) 100 mM NMDG	S6
Table S2.	pK_D of fluorescent ligand **13h** in buffers containing Na^+ and Gpp(NH)p or NMDG	S6
Figure S5.	HPLC chromatograms for compounds **13g** and **13h**	S7
Figure S6.	Fluorescence activated cell sorting (FACS) data for the generation of Flp-In CHO cells stably expressing SNAP-NOPr	S7

Table S1. Structure and opioid receptor affinity of selective NOPr antagonist SB612111. SB612111 was used to determine non-specific binding of fluorescent ligands **13a-h** in fluorescence microscopy applications and **13g-h** in TR-FRET based assays. All data is from Spagnolo et al.,[1].

	pK_i ± SEM (K_i)				pK_B (K_B)
Structure	CHOhNOPr	CHOhMOPr	CHOhDOPr	CHOhKOPr	GTPγ[^{35}S]
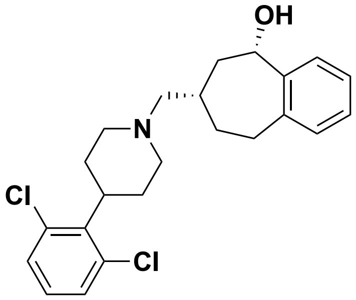	9.80 ± 0.08 (0.16 nM)	<6.0 (<1000 nM)	<5.3 (<5000 nM)	<6.0 (<1000 nM)	9.70 (0.20 nM)

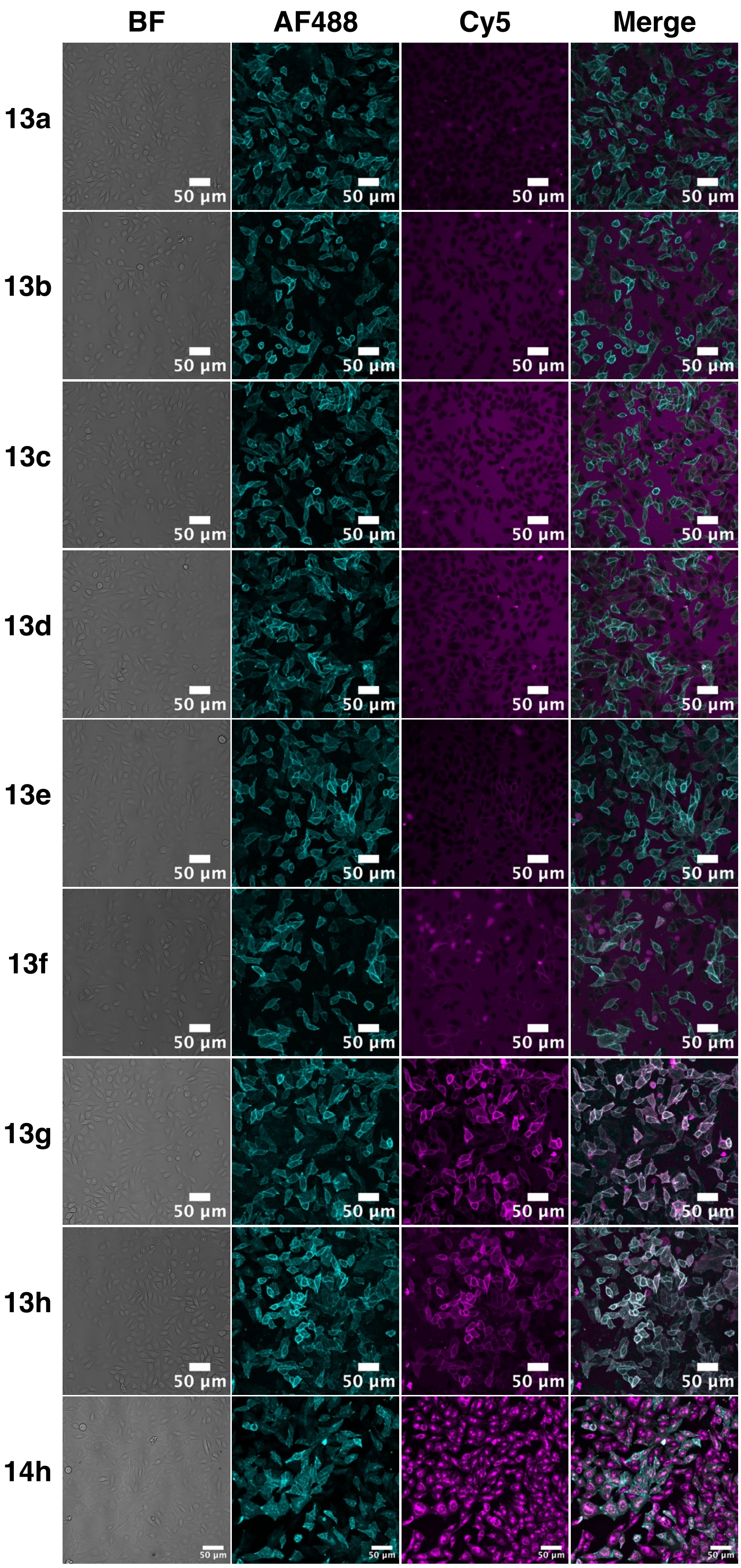

Figure S1. Single time point confocal images of live FlpIn CHO cells stably expressing SNAP-NOPr. Cells were labelled with SNAP-AF488 (cyan) to visualize SNAP-NOPr expression and pre-incubated for 30 minutes with fluorescent ligands **13a-h** and **14h** (magenta) to visualize fluorescent ligand binding. Significant membrane localized SNAP-NOPr expression was seen for all experiments (AF488 channel) with fluorescent ligands **13a-d** showing minimal labelling and fluorescent ligands **13e-h** and **14h** colocalization between AF488 and Cy5 channels and fluorescent ligand binding was completely displaced by SB612111 suggesting NOPr-specific labelling.

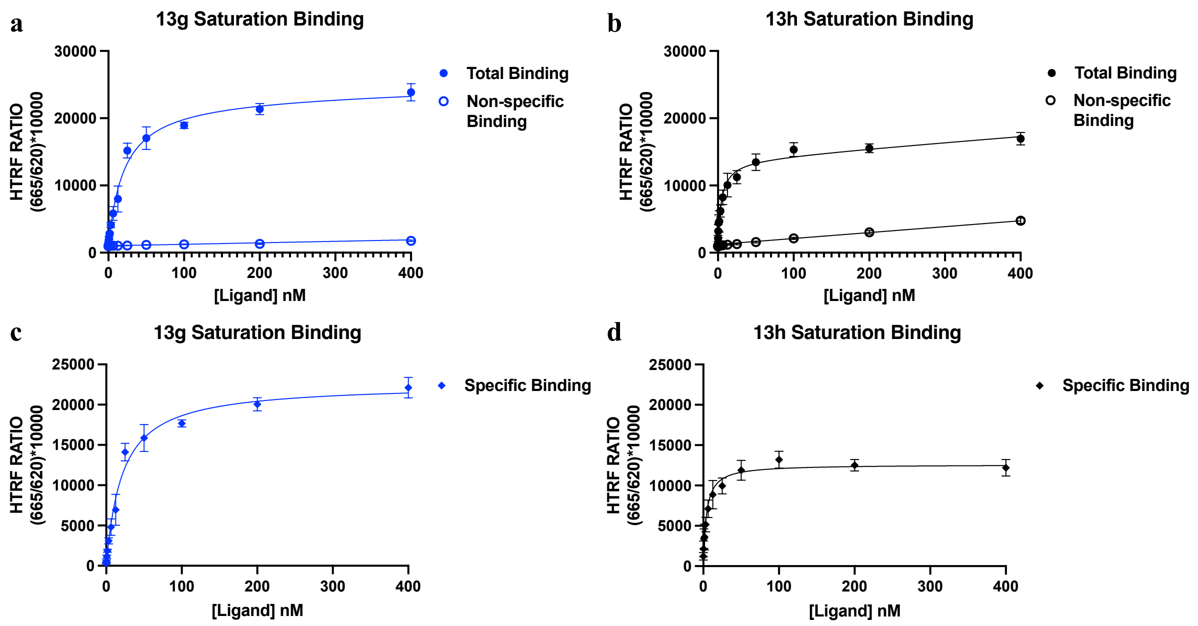

Figure S2. TR-FRET binding experiments to characterise the affinity of fluorescent ligand 13g and 13h to the NOPr. a) **& b**) Equilibrium saturation binding experiments assessing the binding of increasing concentrations of **13g-h** (0-400 nM) to Lumi4-Tb SNAP-NOPr-CHO membranes to determine the affinity. For a) **13g** and b) **13h** the homogenous TR-FRET (HTRF) ratio ((acceptor λ_{em} (665 nM) /donor λ_{em} (620 nM) × 10000) is plotted against ligand concentration (nM) for the total binding (solid blue circle) and non-specific binding (NSB) (hollow blue circle). cSpecific binding curves (total – non-specific) are shown for fluorescent ligand **13g** and **13h** in (c) and (d) respectively. All data represents the mean ± SEM of n=3 experiments performed in duplicate.

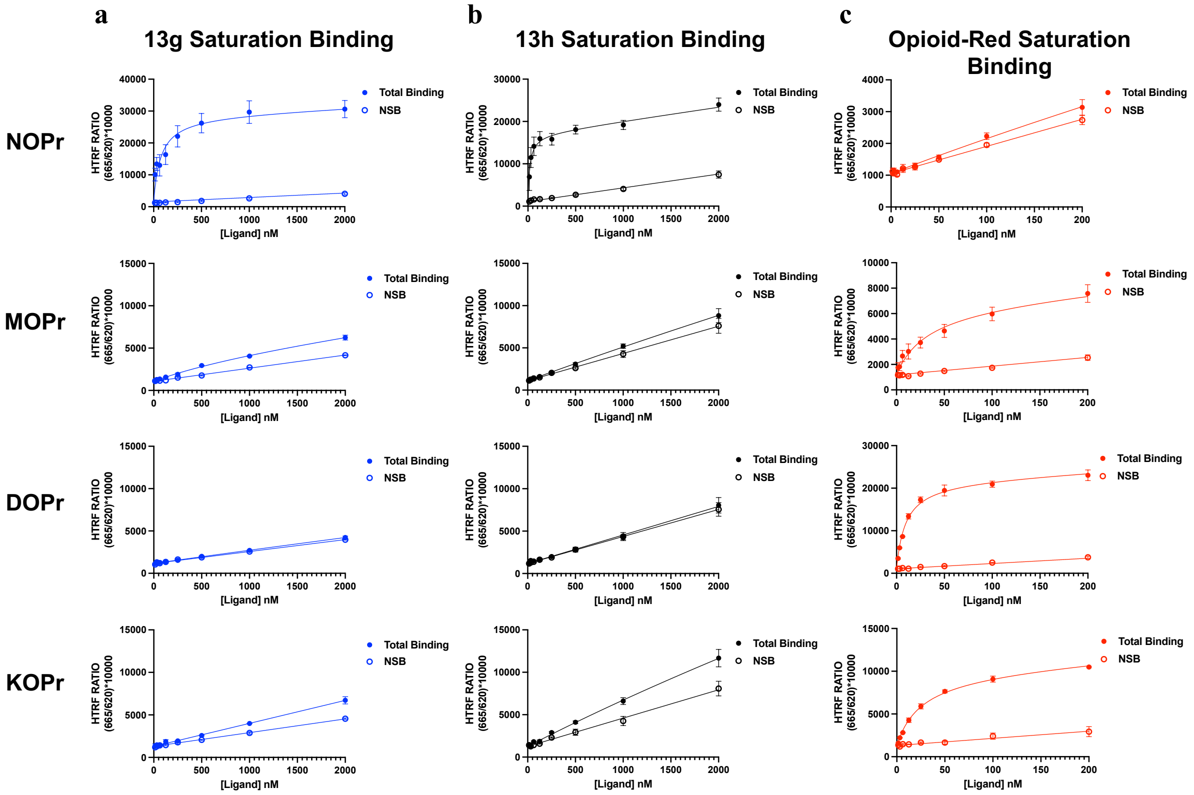

Figure S3. TR-FRET equilibrium saturation binding experiments to determine the selectivity of fluorescent ligands 13g-h against the NOPr; MOPr; DOPr and KOPr. The binding of a) **13g** (0 - 2000 nM) b) **13h** (0 - 2000 nM) and c) naltrexone based opioid red antagonist (Revvity, 0-200 nM) to membranes prepared from Lumi4-Tb-SNAP-NOPRr; Lumi4-Tb-SNAP-MOPr; Lumi4-Tb-SNAP-DOPr; Lumi4-Tb-SNAP-KOPr Flp-In CHO cells was measured after 2 hours at 37 °C. The HTRF ratio x 10000 is plotted against ligand concentration (nM) for the mean± SEM of n=3 experiments.

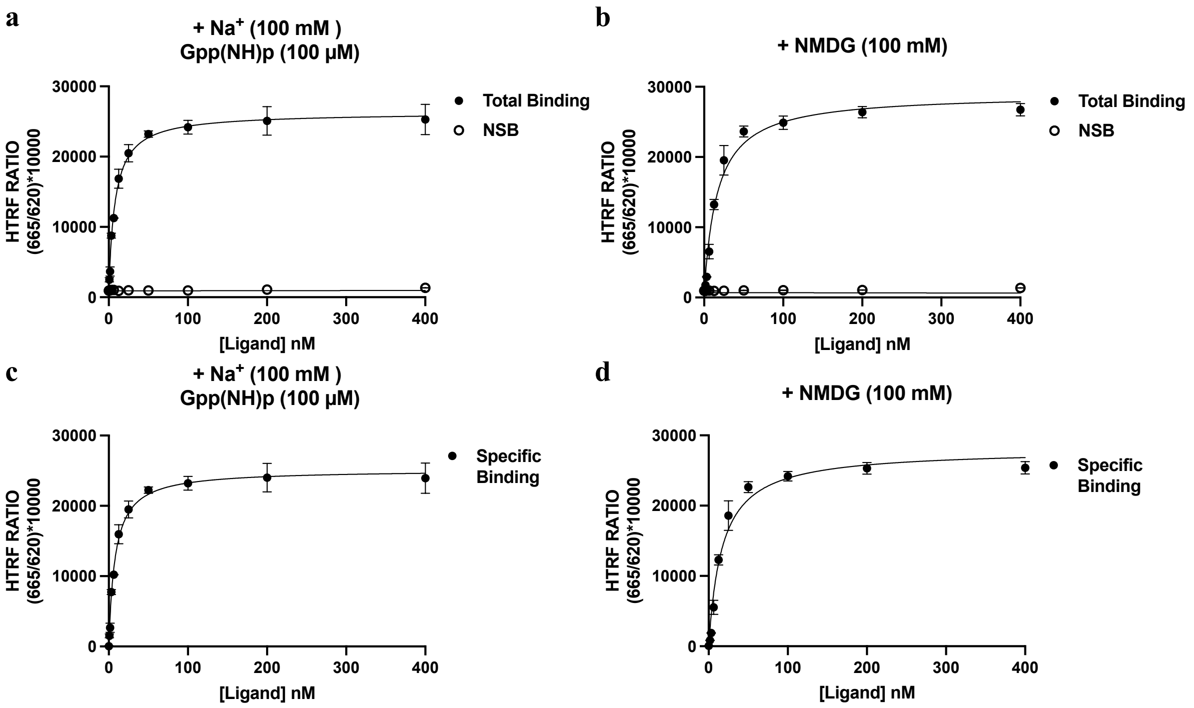

Figure S4. TR-FRET saturation binding experiments to characterize the affinity of fluorescent ligand 13h in buffers containing a) 100 mM Na+ and 100 mM Gpp(NH)p or b) 100 mM NMDG. The binding of increasing concentrations of **13h** (0-400 nM) to Lumi4-Tb SNAP-NOPr-CHO membranes in the presence (non-specific)(black circle) and absence (total-binding) (hollow circle) of SB612111 was assessed after 2 hrs at 37°C. The homogenous TR-FRET (HTRF) ratio ((acceptor λem (665 nM) /donor λem (620 nM) × 10000) is plotted against ligand concentration (nM) for each condition. Specific binding curves (total – non-specific) (black circle) are shown for **13h** in both c) Na^+ and Gpp(NH)p and d) NMDG condition. All data represent the mean ± SEM of n=3 experiments performed in duplicate.

Table S2. pKD of fluorescent ligand 13h in buffers containing Na+ and Gpp(NH)p or NMDG. Data represents the mean ± SEM of n=3 eperiments performed in duplicate.

	+ Na^+ and Gpp(NH)p	+ NMDG
**13h Saturation pK_D (K_D)**	8.08 ± 0.10 (8.3 nM)	7.77 ± 0.04 (17.1 nM)

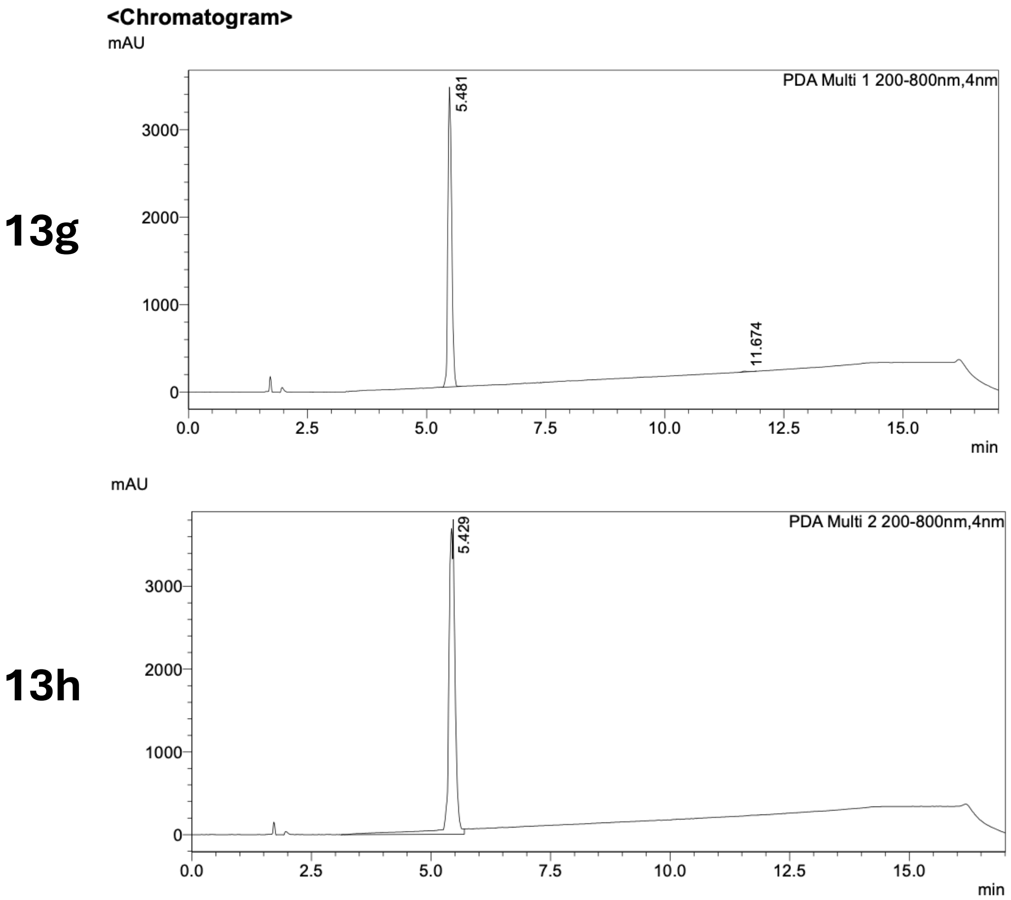

Figure S5. HPLC chromatograms for compounds **13g** and **13h**. Samples **13g** and **13h** were analyzed as described in the experimental using method B and confirmed to be >99% pure.

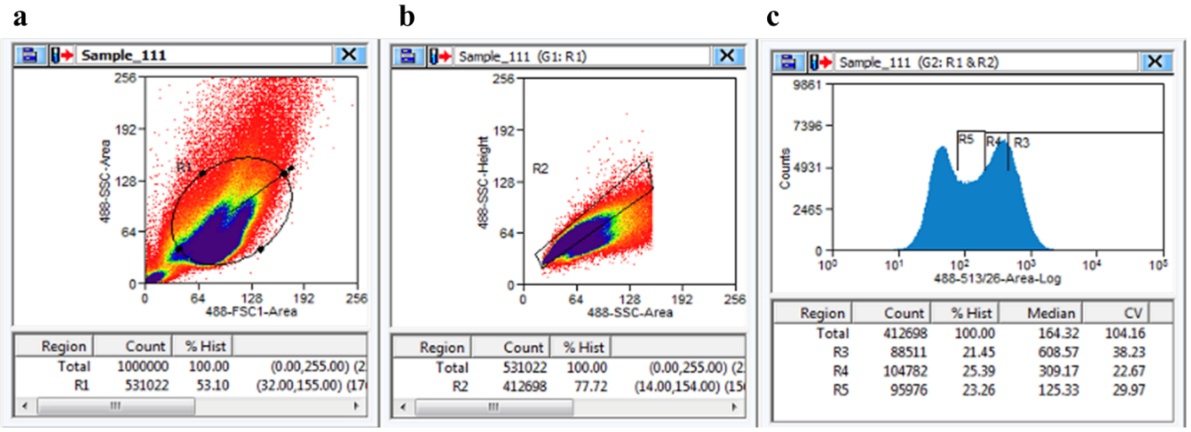

Figure S6. Fluorescence activated cell sorting (FACS) data for the generation of Flp-In CHO cells stably expressing SNAP-NOPr. Flp-In CHO cells expressing SNAP-NOPr were labelled using SNAP-alexafluor-488 (AF488) a) Forward scatter area (FSC-A) vs side scatter area (SSC-A) plot of the total cell population with gate R1 indicating the main cell population excluding debris b) Cells in R1 were further gated (R2) to exclude doublets using AF488 fluorescence height vs fluorescence area. c) Histogram of AF488 fluorescence for cells in R2 showing three expression subpopulations (low (R5) medium (R4) and high (R3)). Single cell clones from the high expression gate (R3) were used in all subsequent experiments.

REFERENCES

(1) Rizzi, A.; Gavioli, E. C.; Marzola, G.; Spagnolo, B.; Zucchini, S.; Ciccocioppo, R.; Trapella, C.; Regoli, D.; Calò, G. Pharmacological Characterization of the Nociceptin/Orphanin FQ Receptor Antagonist SB-612111 [(–)-cis-1-Methyl-7-[[4-(2,6-dichlorophenyl)piperidin-1-yl]methyl]-6,7,8,9-tetrahydro-5 H-benzocyclohepten-5-ol]: In Vivo Studies. *The Journal of Pharmacology and Experimental Therapeutics* **2007**, *321* (3), 968-974. DOI: https://doi.org/10.1124/jpet.106.116780.
